# Ultrastructural Imaging of Activity-Dependent Synaptic Membrane-Trafficking Events in Cultured Brain Slices

**DOI:** 10.1016/j.neuron.2020.09.004

**Published:** 2020-12-09

**Authors:** Cordelia Imig, Francisco José López-Murcia, Lydia Maus, Inés Hojas García-Plaza, Lena Sünke Mortensen, Manuela Schwark, Valentin Schwarze, Julie Angibaud, U. Valentin Nägerl, Holger Taschenberger, Nils Brose, Benjamin H. Cooper

**Affiliations:** 1 Department of Molecular Neurobiology, Max Planck Institute of Experimental Medicine, 37075 Göttingen, Germany; 2 Georg August University School of Science, Georg August University Göttingen, 37073 Göttingen, Germany; 3 Göttingen Graduate School for Neurosciences, Biophysics, and Molecular Biosciences, 37077 Göttingen, Germany; 4 University of Bordeaux, CNRS, Interdisciplinary Institute for Neuroscience, IINS, UMR 5297, F-33000 Bordeaux, France; 5 Cluster of Excellence “Multiscale Bioimaging: from Molecular Machines to Networks of Excitable Cells” University of Göttingen, 37073 Göttingen, Germany

**Keywords:** synapse, synaptic vesicle, active zone, exocytosis, endocytosis, Electron microscopy, Electron tomography, optogenetics, High-pressure freezing, Flash-and-freeze

## Abstract

Electron microscopy can resolve synapse ultrastructure with nanometer precision, but the capture of time-resolved, activity-dependent synaptic membrane-trafficking events has remained challenging, particularly in functionally distinct synapses in a tissue context. We present a method that combines optogenetic stimulation-coupled cryofixation (“flash-and-freeze”) and electron microscopy to visualize membrane trafficking events and synapse-state-specific changes in presynaptic vesicle organization with high spatiotemporal resolution in synapses of cultured mouse brain tissue. With our experimental workflow, electrophysiological and “flash-and-freeze” electron microscopy experiments can be performed under identical conditions in artificial cerebrospinal fluid alone, without the addition of external cryoprotectants, which are otherwise needed to allow adequate tissue preservation upon freezing. Using this approach, we reveal depletion of docked vesicles and resolve compensatory membrane recycling events at individual presynaptic active zones at hippocampal mossy fiber synapses upon sustained stimulation.

## Highlights

• High-pressure freezing of cultured brain tissue without cryoprotectants for EM
• Flash-and-freeze functional EM of identified synapses in complex neural circuits
• Direct correlation of activity-induced functional states and ultrastructure
• Dissection of the spatiotemporal organization of endocytosis in identified synapses

## Graphical Abstract

**Figure:**
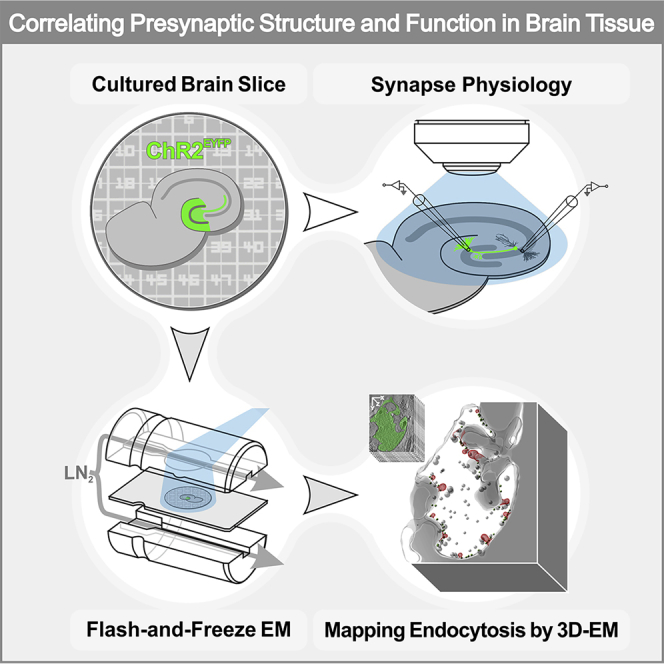

## Teaser

Imig et al. present an experimental workflow for electrophysiological and light-stimulation-coupled high-pressure freezing and electron microscopy to be performed under near-identical conditions in brain tissue cultures. Demonstrating proof of principle, they capture vesicle pool changes and map endocytic events in hippocampal mossy fiber boutons with high spatiotemporal resolution during defined activity states.

## Introduction

Distinct synapse types in the mammalian brain differ substantially with respect to key functional properties, such as transmitter release probability (P_r_), postsynaptic sensitivity, or synaptic plasticity. In this regard, two major open questions in synapse biology are whether distinct ultrastructural synaptic features contribute to or determine fundamentally different functional synapse properties, and how such ultrastructural features are linked to distinct molecular machines and pathways in health and disease (Brose et al., 2019; Kaeser and Regehr, 2017; Neher and Brose, 2018; Xu-Friedman and Regehr, 2004). Addressing these issues experimentally is inherently difficult, as it requires the use of an experimental system where electrophysiological recordings and fixation for electron microscopy (EM) studies can be performed under identical conditions, allowing direct comparisons of functional and ultrastructural data—ideally in a tissue context.

This is particularly challenging with regard to presynapses, where synaptic vesicle (SV) fusion occurs at millisecond timescales and endocytosis operates at millisecond-to-second rates. Aldehyde-based fixation for large-scale 3D EM applications of large tissue blocks are essential to preserve neuronal ultrastructure, e.g., for the study of brain connectivity (Motta et al., 2019), structural changes of axons and dendrites in long-term plasticity (Chirillo et al., 2019; Kuwajima et al., 2020), or sub-synaptic relationships between active zone (AZ) transmitter release sites and vesicle pools in complex synapses (Rollenhagen et al., 2007; Sätzler et al., 2002; Xu-Friedman et al., 2001). However, the relatively slow diffusion of fixatives into tissue may alter presynaptic ultrastructure (Korogod et al., 2015; Maus et al., 2020) or even trigger vesicle fusion (Smith and Reese, 1980). Accordingly, accurate and direct correlations between ultrastructural information (e.g., the number of docked SVs at AZs) and defined synaptic activity states (e.g., short-term plasticity states) have been elusive.

To link ultrastructural observations with defined functional states, near-native preservation of neuronal ultrastructure and a temporal frame of reference are required. Different cryo-fixation techniques have been developed to achieve this, including electrical-stimulation-coupled “slam freezing” (Heuser and Reese, 1981; Heuser et al., 1979), “zap-and-freeze” (Kusick et al., 2020), and “flash-and-freeze” optical stimulation of light-gated-ion-channel-expressing neurons with rapid cryofixation methods such as high-pressure freezing (HPF; Kittelmann et al., 2013; Watanabe et al., 2013a, 2013b). Corresponding studies showed compellingly that morphologically docked vesicles are functionally primed vesicles of the readily releasable pool (RRP) and that distinct modes of endocytosis with different kinetics operate in a variety of different systems, such as frog neuromuscular junctions (Heuser and Reese, 1981), *Caenorhabditis elegans* neuromuscular synapses (Kittelmann et al., 2013; Watanabe et al., 2013a), and dissociated mouse hippocampal neurons (Watanabe et al., 2013b).

Cell-specific synaptic activation of genetically identified neurons in brain circuits requires the use of optogenetics. However, applying optogenetics-based “flash-and-freeze” EM technology to brain tissue is not trivial. The objective of rapid freezing is to immobilize tissue water in a vitreous state and hence avoid ice crystal formation, which perturbs cellular ultrastructure. While thin samples, such as synaptosome preparations (Fernández-Busnadiego et al., 2010) or monolayer cultures (Tao et al., 2018), can be vitrified by plunge-freezing technologies, thicker samples, such as brain slices, require HPF (Dubochet, 1995). Further, satisfactory sample vitrification of acutely dissected or cultured brain sections typically requires the addition of external cryoprotectants (Dubochet, 1995; McDonald et al., 2007), such as bovine serum albumin (BSA; Imig et al., 2014), polyvinylpyrrolidone (PVP; Weil et al., 2017), sucrose (Zuber et al., 2005), polysaccharides (Fernandez-Fernandez et al., 2017; Studer et al., 2014), or 1-hexadecene (Korogod et al., 2015). The potential effects of external cryoprotectants on neuronal function are difficult to predict, but most cryoprotectants are poorly compatible with parallel electrophysiological experiments, so that functional control experiments have to be performed in artificial cerebrospinal fluid (ACSF) alone, i.e., under conditions that do not match the conditions of parallel EM analyses.

**Figure 1:**
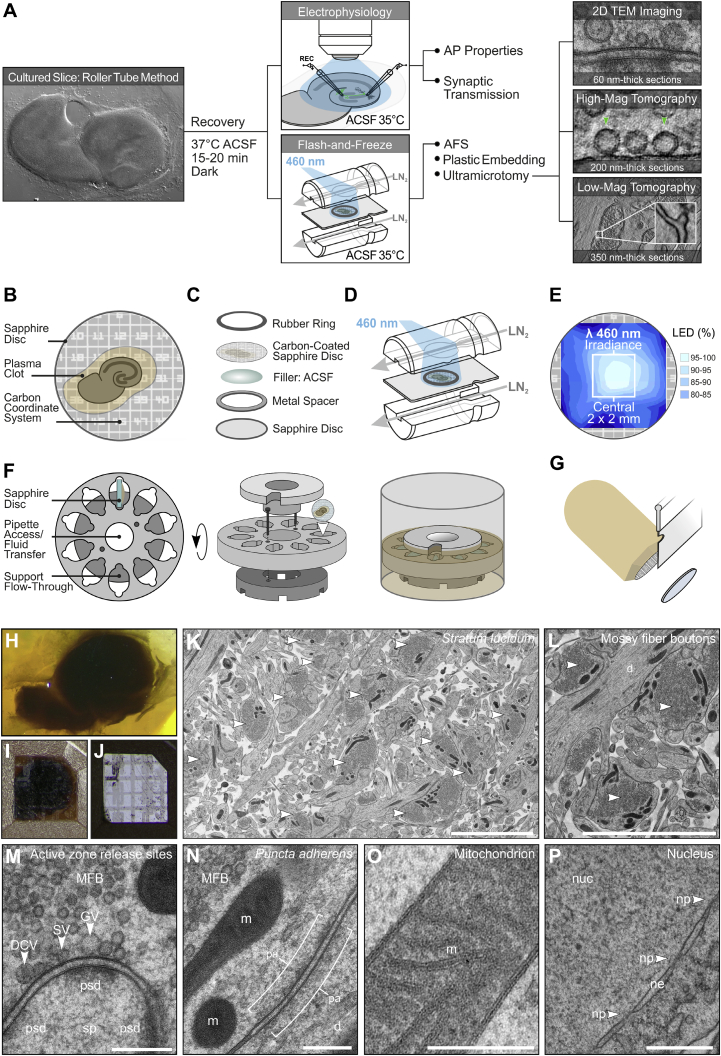
Workflow for Flash-and-Freeze EM of Organotypic Hippocampal Slice Cultures (A) Workflow for correlated electrophysiological and ultrastructural analyses. (B) Schematic of the modified Gähwiler slice culture system. (C) Schematic of the sapphire disc assembly. (D) On-sapphire slices frozen in a Leica EM ICE after exposure to blue (460 nm) light. (E) Factory calibration of the 460 nm LED illumination intensity (schematic adapted from Leica calibration data). (F) Schematic of the configuration of a custom-built aluminum sapphire disc revolver for AFS. (G) Carbon coordinates remain on the surface of the polymerized block following removal of the disc. (H–J) A plastic embedded organotypic slice viewed by transmitted light before (H) and after (I) blockface trimming. The carbon used to guide trimming is visible in reflected light (J). (K and L) Transmission electron micrographs acquired in CA3 *stratum lucidum.* White arrowheads, mossy fiber (MF) terminals. (M) A high-magnification view of a MF AZ. (N) *Puncta adherens* onto a dendrite. (O and P) Vitrified samples are characterized by well-preserved mitochondrial (O) and nucleus morphology (P). ACSF, artificial cerebrospinal fluid; AFS, automated freeze substitution; AP, action potential; d, dendrite; DCV, dense-core vesicle; GV, giant vesicle; m, mitochondrion; nuc, nucleus; ne, nuclear envelope; np, nuclear pore; pa, *puncta adherens*; PSD; postsynaptic density; sp, spine; SV, synaptic vesicle; TEM, transmission electron microscopy. Scale bars: 5 μm (K and L); 200 nm (M–O); 500 nm (P). See also Figures S1 and S2.

In essence, complementary, cell-specific electrophysiological, ultrastructural, and—ultimately—molecular analyses of synapses in mammalian brain tissue require a versatile experimental system. In the study presented here, we established such an experimental system, one that employs the same conditions for all relevant functional and ultrastructural readouts (Figure 1A; STAR Methods). Specifically, our workflow combines mouse genetics, to drive the expression of the light-gated ion channel Channelrhodopsin-2 (ChR2) in specific neuronal subpopulations, with electrophysiology techniques and “flash-and-freeze” EM of synapses in organotypic tissue explant cultures (Figure 1A).

To demonstrate the potential of our approach, we focused on the complex hippocampal mossy fiber (MF) synapses, which connect hippocampal dentate gyrus granule cells (GCs) and *cornu ammonis* subfield 3 (CA3) pyramidal cells. This synapse exhibits a very low initial P_r_ along with distinct presynaptic short-term and long-term plasticity features (Nicoll and Schmitz, 2005). MF synapses are inherently difficult to recapitulate in dissociated neuron cultures (Rost et al., 2010; Tong et al., 1996), but retain most of their anatomical and functional features and their target cell specificity in organotypic slices (Frotscher et al., 2007; Maus et al., 2020; Mori et al., 2004; Studer et al., 2014). Structurally, hippocampal mossy fiber boutons (hMFBs) form large presynaptic terminals and up to 30–45 AZ contact sites with complex postsynaptic spines of CA3 pyramidal cells (Chicurel and Harris, 1992; Rollenhagen et al., 2007), and every CA3 pyramidal cell receives up to 50 MF inputs (Amaral et al., 1990). Vesicle fusion and endocytosis in an entire MF terminal can be monitored at high temporal resolution with direct presynaptic patch-clamp recordings (Delvendahl et al., 2016; Hallermann et al., 2003; Midorikawa and Sakaba, 2017). However, morphological evidence of distinct modes of endocytosis and spatial information on the location of vesicle membrane fusion and retrieval events with respect to individual AZ release sites of MF synapses has been lacking.

## Results

### A Modified Mouse Hippocampal Slice Culture System for Flash-and-Freeze Experiments

To reliably achieve good ultrastructural preservation of brain tissue in ACSF after HPF, we adopted the roller-tube organotypic slice culture system (Gähwiler, 1981, 1984), growing mouse hippocampal slices directly on 6 mm sapphire disc freezing substrates (Figure 1B; STAR Methods), which are compatible with most HPF devices, such as the Leica EM ICE we used. Although acute brain slices are widely used for neuronal connectivity and network studies, we opted against them based on critical methodological considerations concerning key aspects of data quality and interpretation:

Acute cortical vibratome slice preparations based on well-established protocols for slice electrophysiology experiments (Bischofberger et al., 2006) are only compatible with HPF when the focus lies on synaptic ultrastructure at rest (Korogod et al., 2015; Studer et al., 2014), and it is problematic to adopt this system directly for ultrastructural studies of optogenetically activated synapses. Even with optimized slicing procedures, severe tissue damage, including axon transection and the presence of functionally compromised synaptic boutons close to the slice surface (<10–20 μm), are observed (Bischofberger et al., 2006; Korogod et al., 2015; Studer et al., 2014). In view of this, and without flanking connectomics approaches, it is essentially impossible to determine whether EM-imaged synapses are axonally connected to cell somata. It is difficult to predict—or experimentally assess—how, for example, axonally “disconnected” hMFBs compare to intact ones upon optogenetic stimulation. To somewhat reduce the risk posed by these issues, ACSF has to be supplemented with cryoprotectants to achieve adequate ultrastructural preservation of synapses deeper within the acute slice (>10 μm; Borges-Merjane et al., 2020; Dubochet, 1995; Studer et al., 2014). This, however, creates another major problem, as corresponding electrophysiological control experiments, which are required to validate stimulation protocols, can typically not be performed reliably in the presence of cryoprotectants. Thus, electrophysiological validation data, obtained in ACSF alone, and ultrastructural data, obtained in the presence of cryoprotectants, are not directly relatable.

Organotypic slice culture systems, on the other hand, are ideally compatible with the HPF technology since the tissue recovers from the slicing trauma and synapses closer to the surface can be imaged. In view of this key advantage, and because they recapitulate most functional and structural features of the synaptic connections (Maus et al., 2020; Mori et al., 2004, 2007; Studer et al., 2014), we chose organotypic hippocampal slice cultures for our study. Instead of interface cultures on membrane inserts (Frotscher et al., 2007; Maus et al., 2020; Siksou et al., 2009; Stoppini et al., 1991; Studer et al., 2014), we implemented the roller-tube system (Gähwiler, 1981), where glass coverslips or sapphire discs can serve as the culture substrate for brain tissue cultured in a plasma clot. In contrast to this, interface slices are free floating so that their position relative to the optical axis of the stimulation light and to the transparent sapphire disc cannot be controlled during HPF. Manipulations designed to minimize the latter issue, e.g., by increasing slice thickness or reducing freezing cavity depth, can only improve freezing quality at the risk of tissue compression.

Our system allows the inversion of the sapphire disc prior to freezing without detachment of the tissue (Figure 1C). This maximizes the exposure of cells at the sapphire-tissue interface to the light stimulus in the freezer (Figure 1D). The loading of the freezing assembly is performed under red-light conditions (STAR Methods). Briefly, a sapphire disc is submerged in ACSF and an aluminum spacer ring is placed on top. The tissue is never directly manipulated with tools prior to freezing, as the sapphire disc carrying the slice is simply carefully taken out of the recovery chamber with forceps and inverted on top of the ring to close the freezing “sandwich.” We used a thin carbon coordinate system (<4 nm) to facilitate targeted ultramicrotomy of hippocampal subregions and to allow their relative position to be approximately correlated with the calibrated light intensity in the HPF chamber (Figure 1E; STAR Methods). Frozen explants on discs are then cryosubstituted using automated freeze substitution (Figure 1F) and embedded in epoxy resin for ultramicrotomy (Figures 1G–1J) and EM (Figures 1K–1P). Our experimental workflow allows the monitoring of tissue depth for each individual section and therefore to restrict analyses to synapses within 10 μm from the sapphire disc surface (STAR Methods). In this region, the specimens exhibited excellent cryopreservation in ACSF alone (Figures 1M–1P), and we consistently found high densities of hMFBs in CA3 *stratum lucidum* (Figures 1K–1M). We focused analyses exclusively on AZs that contacted spines and not dendrites, as the latter contain *puncta adherentia* that are often difficult to discriminate from AZs (Figure 1N).

We first determined the ultrastructural organization of vesicle pools after HPF in wild-type slices frozen in ACSF at rest and in the presence of glutamate receptor blockers and 1 μM tetrodotoxin (TTX; Figures S1A–S1O). Analysis of high-resolution electron tomograms from AZ profiles of the two main glutamatergic synapses in the hippocampal circuitry, i.e., GC-CA3 pyramidal cell MF (Figures S1A–S1H; Table S1A) and CA3-CA1 pyramidal cell Schaffer collateral (Figures S1I–S1O; Table S1B) synapses, revealed that the general distribution of vesicles is comparable to that seen in interface organotypic slices frozen in culture medium and in acute slices prepared with a tissue chopper (Maus et al., 2020). In particular, we confirmed that hMFBs contained all key morphological vesicle classes that are characteristic of this synapse type (Figure 1M), i.e., small SVs (diameter <60 nm), dense-core vesicles, and larger or “giant” vesicles (GVs; diameter >60 nm; Henze et al., 2002; Maus et al., 2020; Rollenhagen et al., 2007). In comparison to frozen tissue from interface cultures (Maus et al., 2020; Studer et al., 2014), we noticed more visible extracellular space in our culture system (not quantified; Figure 1K). This can be attributed to the fact that roller-tube slices thin out during the culture period, typically to a few cell layers (Gähwiler, 1981). We further observed in initial experiments that shifting the freezing cavity depth from 200–100 μm reduced the extracellular space (Figures S1P and S1Q), indicating that compression of brain tissue alters the proportion of extracellular space. Accordingly, we chose 150 μm spacer rings as a compromise, which provided adequate ultrastructural preservation without tissue compression.

In summary, we show that cultured brain slices can be rapidly frozen without external cryoprotectants. All functional and morphological experiments were performed at near-physiological temperatures (35°C).

### Calibrating Functional Properties for EM Experiments

**Figure 2:**
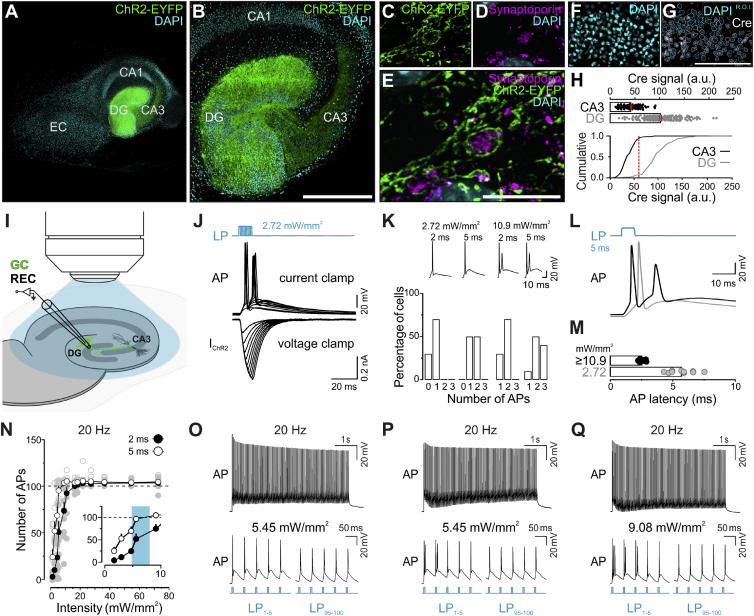
Functional Characterization of Granule Cell (GC) Firing Properties in Dock10-Cre;Ai32 Slices (A–E) ChR2-EYFP expression (green) in the dentate gyrus (DG) and in mossy fibers to the CA3. Cell nuclei visualized by DAPI (cyan; A and B). ChR2-EYFP (green) in synaptoporin-positive MFBs (magenta) in the CA3 (C–E). (F–H) Percentage of Cre-recombinase positive (G, white) DAPI-stained nuclei (F, cyan) and (G, cyan outlines) in the DG. Scatterplot (H, top) and cumulative distribution (H, bottom) indicating the quantification of Cre-signal in CA3 pyramidal (CA3, “background signal”) and DG granule cells (DG; “real signal”). More than 91% of GCs express (red line) Cre above threshold (H, CA3, n = 3 slices; 40.61 a.u. ± 6.28; CA3, n = 3; 83.67 a.u. ± 4.67). (I) Experimental setup for measuring light evoked GC action potential (AP) properties (J–Q). (J) Exemplary recording in which increasing light pulse (LP) duration (1 to 10 ms) triggered zero to two APs (top representation) and a ChR2-mediated current (I_ChR2_) of increasing size and duration (bottom representation). (K), Summary data on AP firing in response to 2 ms or 5 ms LPs at 2.72 and 10.9 mW/mm^2^ (n = 10 cells). (L and M) Latency of APs evoked by a 5 ms LP at 2.72 (5.72 ± 0.32 ms, n = 10) and 10.9 mW/mm^2^ (2.55 ± 0.08 ms, n = 10), respectively, measured from the onset of the LP. (N), AP firing reliability during 100 LP trains (20 Hz) for different light intensities (2 ms, n = 10; 5 ms, n = 10). (O–Q) Exemplary recordings from three different GCs of AP trains in response to 100 × 5 ms LPs at 20 Hz (O), 5.45 mW/mm^2^ (P), and 9.08 mW/mm^2^ (Q). The first (bottom left) and last (bottom right) five APs are shown at an expanded timescale. Scale bars: 500 μm (B); 10 μm (E); 100 μm (G). Error bars indicate mean ± SEM. See also Figures S3 and S4.

We then validated that ChR2-expressing neurons and synapses in cultured hippocampal slices retain their hallmark functional features after development *in vitro*. We generated slice cultures from mice specifically expressing a ChR2^H134R^-enhanced yellow fluorescent protein (EYFP) fusion protein in GCs of the dentate gyrus (Dock10-Cre [Kohara et al., 2014]; Ai32 [Madisen et al., 2012]; Figures 2A–2H) or in glutamatergic forebrain neurons, including GCs (Nex-Cre [Goebbels et al., 2006]; Ai32 mice; Figures S2E–S2T). Biocytin filling of GCs and CA3 pyramidal cells and post hoc labeling with Alexa Fluor-555-coupled streptavidin showed that pre- and postsynaptic specializations of the MF-CA3 projection remain intact in cultured slices (Figures S2A–S2D).

Next, we performed whole-cell patch clamp recordings of CA3 or CA1 pyramidal cells (2 mM external Ca^2+^) and measured excitatory postsynaptic currents (EPSCs) and paired-pulse ratios (PPR) in response to high-frequency electrical stimulation of ChR2-expressing MF (Figures S3A–S3D) or Schaffer collateral axons (Figures S3E–S3G). hMFBs exhibited an initially low P_r_ and strong short-term facilitation, which is characteristic of tonic synapses (Evstratova et al., 2014; Lawrence et al., 2004; Salin et al., 1996), whereas Schaffer collateral synapses (Dobrunz and Stevens, 1997) showed initial facilitation followed by prominent short-term depression, consistent with phasic neurotransmission (Figure S3). These findings indicate that basic functional synaptic properties are preserved in slice culture.

**Figure 3:**
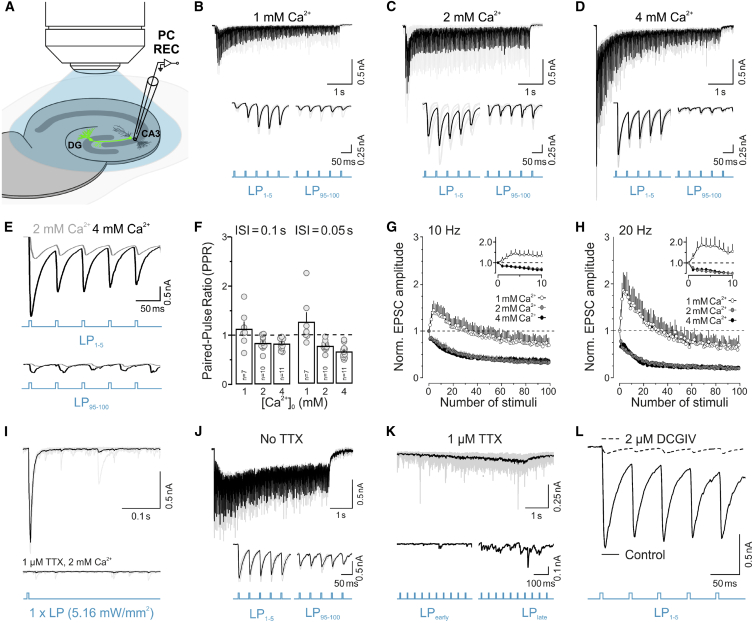
Functional Characterization of CA3 Pyramidal Cell Responses in Dock10-Cre;Ai32 Slices (A) Experimental setup for recording postsynaptic CA3 pyramidal cell (PC) responses (B–L). (B–D) Excitatory postsynaptic currents (EPSCs) in response to 100 × 5 ms light pulses (LPs; 20 Hz, ~5.16 mW/mm^2^) recorded at three different external calcium concentrations, [Ca^2+^]_0_ = 1 mM (B); [Ca^2+^]_0_ = 2 mM (C); and [Ca^2+^]_0_ = 4 mM (D). Traces in (B) and (C) were recorded from the same cell. The first (bottom left) and last (bottom right) five responses are shown with an expanded timescale. (E) EPSC responses to the initial (top) and final (bottom) five stimuli in a 100 LP train (20 Hz, ~5.16 mW/mm^2^) at 2 mM (gray) and 4 mM (black) [Ca^2+^]_0_ shown superimposed. (F) Paired-pulse ratios at different [Ca^2+^]_0_ for two LPs (5.16–9.08 mW/mm^2^) and inter-stimulus intervals (ISI). (G and H) Normalized EPSC amplitudes for 100 LPs (5.16–9.08 mW/mm^2^) at 10 Hz (G) and 20 Hz (H) at 1 mM (white, n = 7 each) 2 mM (gray, n = 10 each), and 4 mM (black, n = 11 each) [Ca^2+^]_0_. (I) EPSCs for a single LP (5.16 mW/mm^2^; [Ca^2+^]_0_ = 2 mM) before (top trace) and after (bottom trace) the application of 1 μM TTX. (J and K) EPSCs in response to 100 LPs (20 Hz; 5.45 mW/mm^2^; [Ca^2+^]_0_ = 2 mM) before (J) and after (K) application of 1 μM TTX. Initial (bottom left) and final (bottom right) responses are shown at expanded timescales. (L) EPSCs in response to five LPs (20 Hz; 5.45 mW/mm^2^; [Ca^2+^]_0_ = 2 mM) before (solid line) and after (dashed line) application of 2 µM DCGIV. Error bars indicate mean ± SEM.

We then characterized the GC-CA3 pyramidal cell MF connection using optogenetic stimulation (Figures 2 and 3). We first determined action-potential (AP) firing properties of ChR2-expressing hippocampal GCs (Figures 2I –2Q). The specific aim was to test the response of individual GCs to light pulses (LPs) applied within a range of intensities that cover the intensity range achieved in the freezing chamber of the Leica EM ICE HPF device (maximum irradiance at the sapphire disc surface between 5.5 and 8.0 mW/mm^2^; STAR Methods), and at slightly lower and higher intensities to account for some potential deviation from these standard values (Figure 1E; STAR Methods). All of these experiments were performed in the presence of glutamate receptor blockers to abolish excitatory synaptic transmission (STAR Methods). In slices from Dock10-Cre;Ai32 animals, single LP (2 ms/5 ms duration) induced AP firing in GCs (Figures 2J–2M) and firing was supported also during trains (100 LPs at 20 Hz; Figures 2N and 2Q) designed to drive sustained vesicle fusion in hMFBs to at least partially deplete the RRP (Barthet et al., 2018). At low light intensities (2.72 mW/mm^2^), 2 ms and 5 ms LPs generally triggered fewer APs than at high intensities (≥10.9 mW/mm^2^; Figures 2J and 2K). At both intensities, 5 ms LPs frequently triggered multiple (two–three) APs (Figures 2K and 2L). Moreover, higher intensities triggered APs with, on average, shorter and less variable latencies (≥10.9 mW/mm^2^, 2.55 ± 0.08 ms versus 2.72 mW/mm^2^, 5.72 ± 0.32 ms; Figure 2M). During repetitive stimulation (100 LPs at 20 Hz) only 5 ms LPs reliably triggered ∼100 APs at intensities ≥5 mW/mm^2^, whereas 2 ms LPs were not sufficient (Figure 2N). For lower intensities (≤5 mW/mm^2^), 5 ms LPs yielded a larger total count of APs (Figure 2N). The likelihood of multiple AP firing, exclusively at the beginning of the train, also increased with increasing light intensities (Figures 2O–2Q), likely due to large initial ChR2-mediated photocurrents (Berndt et al., 2011).

We then measured optically evoked EPSCs in CA3 pyramidal cells in Dock10-Cre;Ai32 slices (Figure 3). In contrast to electrical fiber stimulation experiments, where only a fraction of MFs are activated depending on the stimulation electrode position (Figure S3), full-field light stimulation will stimulate all ChR2-expressing GCs. Moreover, ChR2 expressed in hMFB membranes (Figure 2E) may also be activated, which might lead to AP-independent depolarization of the hMFB. It is very important to note that these experimental conditions had to be specifically designed despite these caveats to mimic the situation in the freezing chamber of the HPF device. Thus, our functional data can be directly related to the EM data, but they cannot be directly compared to those obtained using focal optogenetic stimulation of only a few GC dendrites (Barthet et al., 2018).

We found that at low (1 mM) extracellular Ca^2+^, the pooled postsynaptic responses strongly facilitated during a train of 100 5 ms LPs at 20 Hz (∼5.46 mW/mm^2^; Figure 3B), whereas elevating extracellular Ca^2+^ to 2 or 4 mM gradually increased the initial EPSC amplitudes (Figures 3C–3E; 1 mM Ca^2+^ 0.98 ± 0.28 nA [n = 14 trials]; 2 mM Ca^2+^ 1.87 ± 0.41 nA [n = 20 trials]; 4 mM 4.621 ± 0.38 nA [n = 22 trials]), and reduced the PPR (Figure 3F). Moreover, increasing extracellular Ca^2+^ reduced the degree of facilitation at MF synapses during 10- and 20 Hz stimulations (Figures 3G and 3H). Application of 1 μM TTX to block AP generation completely abolished EPSCs in CA3 pyramidal cells after single LPs (Figure 3I). Also, TTX application during high-frequency trains largely blocked synaptic transmission (Figures 3J and 3K), but we noted an increase in asynchronous-like activity in later phases of the stimulation train (Figure 3K, bottom representation). These findings indicate that the majority of the optically evoked transmitter release is triggered by AP-induced depolarization of the presynaptic membrane, but at least some vesicle fusion events may also occur independently of AP firing in GCs during sustained optogenetic stimulation. Lastly, we confirmed that postsynaptic CA3 pyramidal cell responses were blocked in the presence of the mGluR2/3 receptor agonist DCG-IV (Figure 3L). This finding validates the cell-type-specificity of ChR2-expression in GCs in the hippocampus of Dock10-Cre mice.

In sum, we demonstrate that it is possible to reliably trigger cell-specific neurotransmitter release at hMFBs using optogenetics and that the amount of release is scalable—for example by manipulating external Ca^2+^-concentrations. Our observations of multiple AP firing at high light intensities at the beginning of stimulation trains and the apparent increase in P_r_ in MF synapses in response to optogenetic stimulation in comparison to electrical fiber stimulation are in line with previous reports for other neuron types (Berndt et al., 2011; Zhang and Oertner, 2007). This is a likely result of the relatively slow off-kinetics of the ChR2^H134R^ variant that induces large photocurrents and a long after-depolarization following optically triggered APs (Berndt et al., 2011).

### Long Stimulation Protocols Induce Vesicle Depletion in the Vicinity of AZs

**Figure 4:**
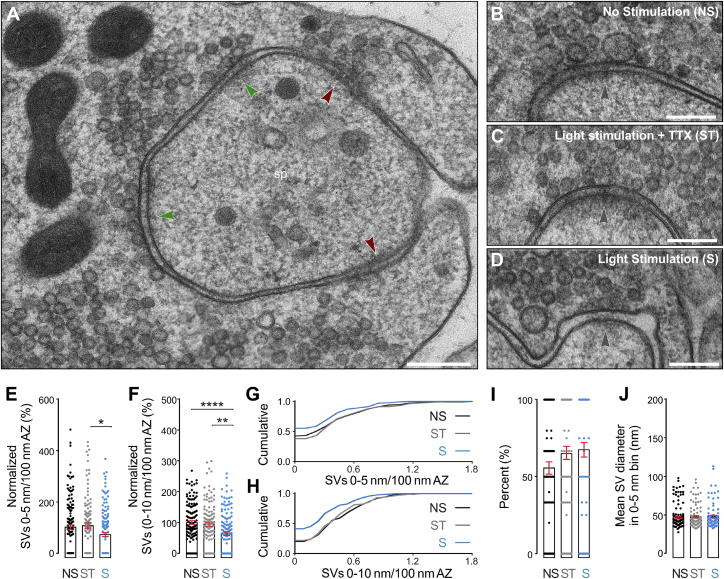
2D Ultrastructural Analysis of Mossy Fiber Synapses from Dock10-Cre;Ai32 Slices after High-Frequency Stimulation (100 × 5 ms LPs at 20 Hz, 2 mM Ca^2+^) (A) Electron micrograph of active zone (AZ) profiles contacting spines (sp). Cross-sections of AZs with defined bilayers (green arrowheads) were analyzed, whereas AZ profiles exhibiting high membrane curvature were excluded (red arrowheads). (B–D) Electron micrographs of AZs in three experimental conditions: NS, no stimulation (B); ST, light stimulation + 1 μM TTX (C); S, light stimulation (D). (E and F) Spatial density of vesicles within 0 to 5 nm (E) and 0 to 10 nm (F) of the AZ per 100-nm AZ length. Values are normalized to the NS control condition. (G and H) Cumulative distribution of vesicles within 0 to 5 nm (G) and 0 to 10 nm (H) of the AZ per 100 nm AZ length. (I) Relative proportion of all vesicles within 0 to 10 nm of the AZ localized within in the 0 to 5 nm bin. (J) Mean diameter of vesicles within 0 to 5 nm of the AZ. NS, 136 vesicles; ST, 105 vesicles; S, 94 vesicles. Scale bars: 200 nm (A–D). Error bars indicate mean ± SEM; ^∗^p < 0.05; ^∗∗^p < 0.01; ^∗∗∗∗^p < 0.0001. NS, three cultures, three slices, 47 MFBs, n = 135 AZs; ST, three cultures, three slices, 39 MFBs, n = 114 AZs; S, three cultures, three slices, 38 MFBs, n = 140 AZs. See also Table S1C and Figure S5.

To induce reliable vesicle fusion at 2 mM Ca^2+^, we applied 100 LPs at 20 Hz (Barthet et al., 2018) prior to HPF fixation using Dock10-Cre;Ai32 slices and a 5 ms pulse duration. Freezing (the time point at which the temperature sensor of the HPF device reaches 0°C) was set to occur 5 ms after the onset of the last LP in the train. We estimated the time point at which the slice at the sapphire-tissue interface reaches 0°C to be additionally delayed by approximately 5 ms (Kusick et al., 2020). Taking into consideration the mean time required for light to evoke an AP (2.55–5.72 ms [after the onset of the LP for light intensities of ≥10.9 mW/mm^2^ and 2.72 mW/mm^2^, respectively; Figure 2M]) and for AP propagation to the presynaptic terminal in hippocampal slice cultures (∼6 ms; Mori et al., 2004), we estimate that stimulated synapses were frozen at <10 ms after the arrival of the last AP in the terminal. To discriminate potential morphological consequences of AP-triggered fusion from those induced by Ca^2+^ influx as a result of ChR2 activation in the terminal, we froze slices from each culture either in the dark (no stimulation, NS), after optical stimulation in the presence of 1 μM TTX (light stimulation + TTX, ST), or after optogenetic stimulation without TTX (light stimulation; S). We first quantified all vesicles (SVs and GVs) in the AZ vicinity in 2D electron micrographs of hMFBs (Figure 4; Table S1C). Due to the relatively high curvature of the presynaptic membrane at spines, only a subset of AZs was included in the analysis for a given MF profile (Figures 4A–4D). Using this approach, we found a tendency toward reduced vesicle numbers within 0–5 nm of the AZ after stimulation (Figure 4E). This reduction was accompanied by a strong depletion of vesicles within 10 nm of the AZ in comparison to both control conditions (Figure 4F). Further, the fraction of AZs without vesicles in the vicinity increased after stimulation (Figures 4G and 4H). We also noticed a slight, but not statistically significant shift toward a higher proportion of vesicles to be “docked” (within 0–5 nm) relative to all vesicles within 10 nm of the AZ after stimulation, irrespective of whether TTX was present or not (Figure 4I; see below). No changes in the size distribution of docked vesicles were observed (Figure 4J).

**Figure 5:**
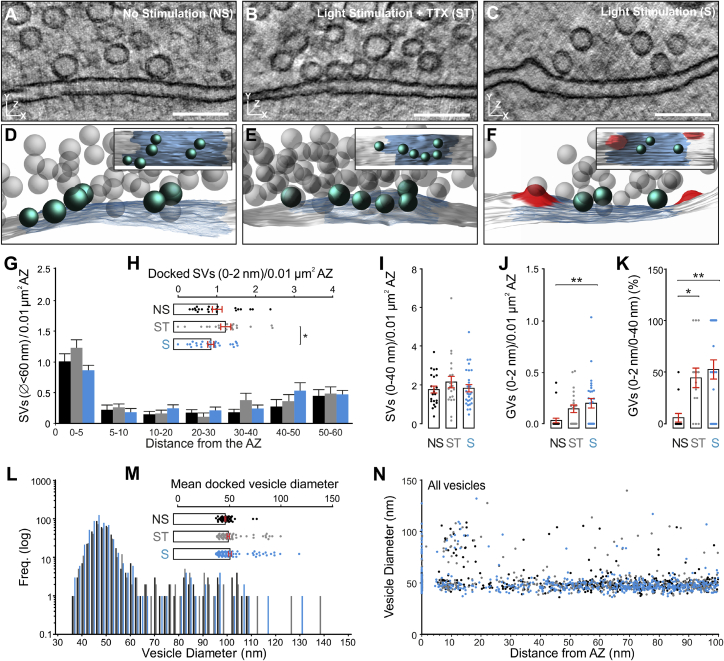
3D Ultrastructural Analysis of Mossy-Fiber-CA3 Synapses in Dock10-Cre;Ai32 Slices after High-Frequency Stimulation (100 × 5 ms LPs at 20 Hz, 2 mM Ca^2+^) (A–C) High-magnification electron tomographic subvolumes of active zones (AZs) for three conditions: NS, no stimulation (A); ST, stimulation in the presence of 1 μM TTX (B); S, stimulation (C). (D–F) 3D models (docked vesicles, turquoise; undocked vesicles, gray; AZ, blue; putative endocytic pits, red) and orthogonal views (insets). (G) Distribution of SVs (Ø < 60 nm) within 60 nm of the AZ normalized to AZ area. (H) Spatial density of docked SVs (0- to 2-nm bin) normalized to AZ area. (I) SV number within 0 to 40 nm of the AZ normalized to AZ area. (J) Spatial density of docked giant vesicles (GVs; Ø >60 nm) normalized to AZ area. (K) Percentage of docked GVs within 0 to 40 nm of the AZ. (L) Vesicle diameter distribution for undocked vesicles within 100 nm of the AZ (2 nm bins). NS, 395 vesicles; ST, 365 vesicles; S, 516 vesicles. (M) Mean diameter of docked vesicles. NS, 103 vesicles; ST, 115 vesicles; S, 108 vesicles. (N) Spatial distribution of vesicles with respect to vesicle diameter. Scale bars: (A–C) 100 nm. Error bars indicate mean ± SEM; ^∗^p < 0.05; ^∗∗^p < 0.01. NS, three cultures, three slices, n = 22 AZs; ST, three cultures, three slices, n = 21 AZs; S, two cultures, three slices, n = 28 AZs. See also Table S1D and Figure S5.

We next performed high-resolution electron tomography to determine the fine organization of SVs and GVs in the vicinity (0–100 nm) of AZs (Figure 5; Table S1D). In these synaptic sub-volumes, stimulated hMFBs exhibited an overall reduction in the number of SVs docked (0–2 nm) and within 5 nm (Figures 5G and 5H), but not within 40 nm of the membrane (Figure 5I). After stimulation, SV docking was reduced in comparison to the ST control, but due to the high variability between the responses at individual AZs to stimulation, the reduction in vesicle docking after stimulation as assessed in high-magnification tomograms was not significant when compared to the NS control, for the given sample size. Interestingly, we observed an increase in the absolute density (Figure 5J) and the relative proportion of docked GVs among all GVs within 40 nm of the AZ (Figure 5K) after stimulation, irrespective of whether TTX was present or not. Moreover, in line with our findings in the 2D analysis, we noticed a tendency to increased numbers of SVs within 0-5 nm of the AZ in the Stim+TTX condition in comparison to NS slices (Figures 5G and 5H). These findings raise the possibility that Ca^2+^ influx through ChR2 into presynapses, though not sufficient to significantly trigger vesicle fusion, may slightly affect SV and GV docking in hMFBs (Chang et al., 2018; Maus et al., 2020) or the stabilization of docked vesicles at the membrane, e.g., by preventing de-priming (He et al., 2017). Lastly, electron tomography enabled us to precisely measure vesicle diameters in 3D (Figures 5L–5N). In line with our findings from the 2D analysis (Figure 4J), no significant changes in the average size of docked vesicles were observed between conditions, despite a tendency toward slightly larger diameters after stimulation (Figures 5L and 5M). This apparent increase in the mean vesicle diameter is likely caused by increased GV docking after stimulation (Figure 5J).

In sum, our findings indicate that optogenetic stimulation, and thereby AP-generation, triggers depletion of docked SVs in hMFBs as it has been shown for other synapse types (Chang et al., 2018; Watanabe et al., 2013a, 2013b). Our data also highlight the critical importance of control experiments with TTX to determine potential AP-independent effects of ChR2 activation in the terminal on SV distribution at AZs—especially with long stimulation protocols.

### Mapping the Spatial Organization of Endocytic Events in hMFBs

We then assessed whether the depletion of docked SVs was paralleled by an increase in local vesicle recycling in hMFBs. Previous functional studies measuring presynaptic hMFB membrane capacitance changes in response to depolarization pulses indicated robust endocytosis and a temperature dependence of its kinetics (Delvendahl et al., 2016; Hallermann et al., 2003). However, the mode and spatial organization of endocytosis in hMFBs are unknown.

**Figure 6:**
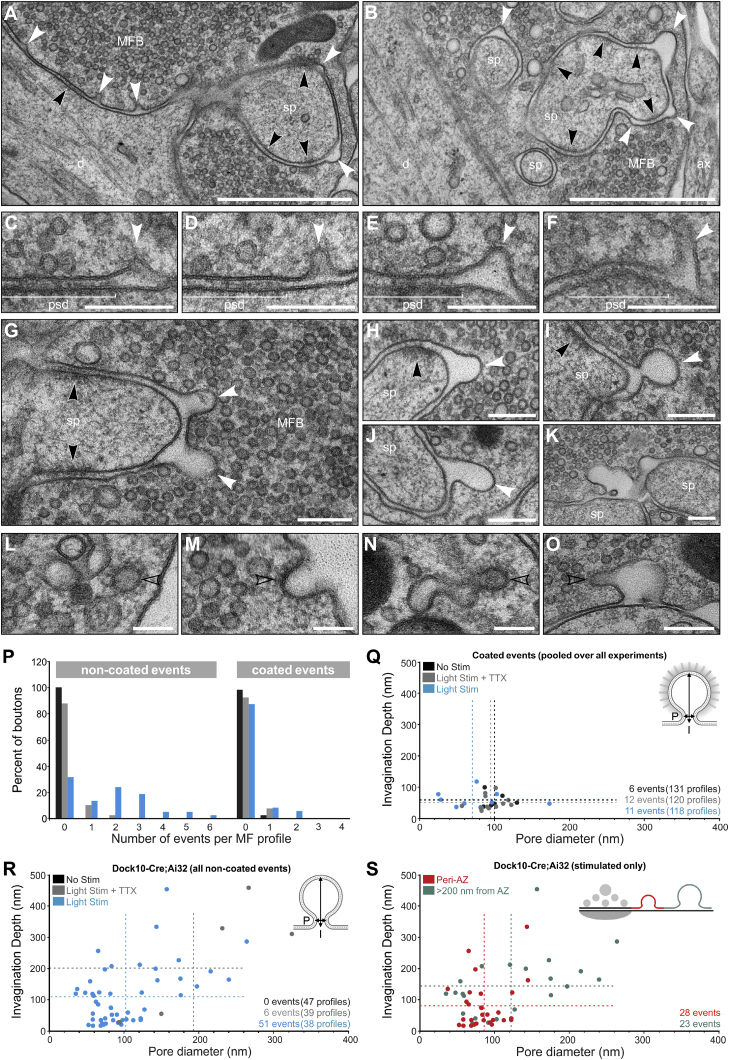
2D Ultrastructural Analysis of Putative Endocytic Profiles in Dock10-Cre;Ai32 Slices after High-Frequency Stimulation (100 × 5 ms LPs at 20 Hz, 2 mM Ca^2+^) (A and B) Transmission electron micrographs of stimulated MFBs. Membrane invaginations are captured at peri-active zonal (AZ) sites (white arrowheads). (C–F) Peri-AZ endocytic pits (white arrowheads). Electron dense material occasionally observed at the invagination lacked the density or periodicity of clathrin coats. (G–K) Bulk-like larger endocytic profiles (white arrowheads). (L–O) Clathrin-coated vesicles (open black arrowheads) in the cytoplasm (L), in the process of forming at the plasma membrane (M), or from elongated organelles (N). Clathrin-coated protrusions emerging from invaginations were only extremely rarely observed (O). NS, 131 MFBs; ST, 120 MFBs; S, 118 MFBs. (P) Relative incidence of coated and non-coated invaginations per MFB profile. NS, no stimulation (black); ST, stimulation in the presence of TTX (gray); S, stimulation (blue). (Q) Relationship between pore diameter (P) and invagination depth (I) for coated intermediates across all experiments analyzed (Pooled data from Dock10-Cre;Ai32 and Nex-Cre;Ai32 slices). Dashed lines indicate respective mean values. (R) Relationship between pore diameter (P) and invagination depth (I) for non-coated (R) endocytic intermediates. Dashed lines indicate respective mean values. (S) Relationship between pore diameter (P) and invagination depth (I) for non-coated (R) endocytic intermediates detected in stimulated MFBs at the peri-AZ (within 0 to 200 nm from AZ; red) and more distal (>200 nm from AZ; green) sites. Dashed lines indicate respective mean values. d, dendrite; mfb, mossy fiber bouton; PSDs, black arrowheads; sp, spine. Scale bars: (A and B), 1 μm; (C–K and O), 200 nm; (L–N), 100 nm. For (P, R, and S): NS, three cultures, three slices, 47 MFBs; ST, three cultures, three slices, 39 MFBs; S, three cultures, three slices, 38 MFBs. See also Table S1E and Figures S6 and S7.

We first analyzed all membrane-invaginations visible in 2D electron micrographs of MF profiles in control and stimulated Dock10-Cre;Ai32 slices (100 × 5 ms LPs at 20 Hz, 2 mM Ca^2+^; Figure 6; Table S1E). We exclusively quantified membrane invaginations rather than large vesicles in the vicinity of AZs to rule out the possibility of including GVs into our analysis, because large vesicular structures are also frequently present in proximity to and docked at MF AZs at rest (Figure 1M; Henze et al., 2002; Maus et al., 2020; Rollenhagen et al., 2007). In stimulated hMFBs, we observed robust signs of activity-induced invaginations, which exhibited a range of different shapes and dimensions (smaller and large, bulk-like invaginations; Figures 6A–6O). The vast majority of these did not exhibit a prominent electron-dense coat (Figures 6A–6K) indicative of clathrin-mediated endocytosis. However, we occasionally captured coated endocytic or vesicular structures (Figures 6L–6O), verifying that clathrin coats are preserved and detectable with our protocol. In general, we only found very few coated membrane-invaginations in non-stimulated and stimulated hMFB profiles, throughout all experiments analyzed as part of this study (Figures 6P and 6Q). For all events (coated [Figure 6Q] and non-coated [Figures 6R and 6S]), we measured the pore diameter and the invagination depth. We found that in contrast to coated events, which on average exhibited a rather uniform invagination depth (Figure 6Q), non-coated events exhibited considerable size variability (Figure 6R). Events within 200 nm of the AZ (peri-AZ) were on average smaller than those at larger distances (>200 nm from AZ; Figure 6S). Although 2D analyses do not permit an entirely accurate mapping of events with respect to AZs, our findings strongly support the notion that bulk-like endocytic events are more prevalent distally in hMFBs. In contrast, only four out of 29 coated events analyzed throughout the entire study were found at the peri-AZ.

**Figure 7:**
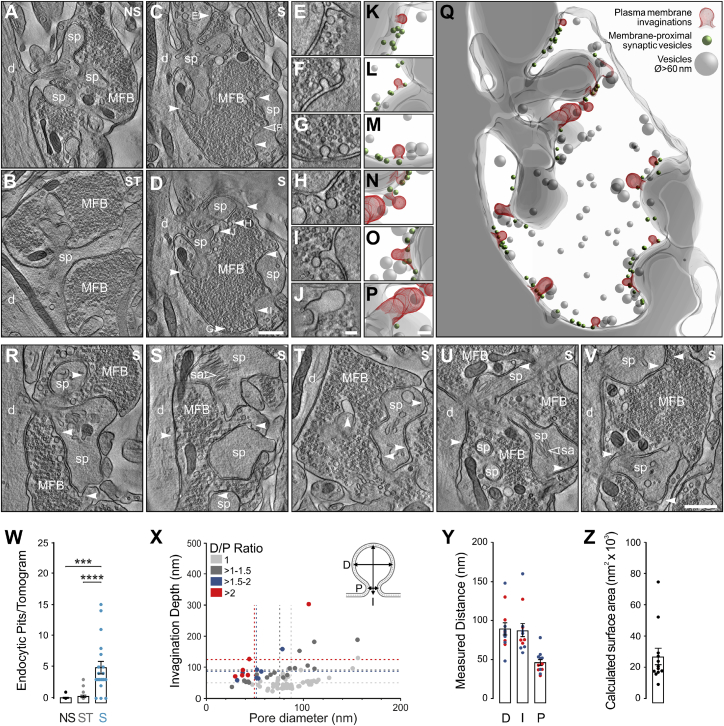
3D Ultrastructural Analysis of Putative Endocytic Profiles in Dock10-Cre;Ai32 Slices after High-Frequency Stimulation (100 × 5 ms LPs at 20 Hz, 2 mM Ca^2+^) (A–D) Low-magnification electron tomographic subvolumes. NS, no stimulation (A); ST, stimulation in the presence of 1-μM TTX (B); S, stimulation (C); tomographic slices from the same MFB (D). Mapping putative endocytic events that are within (white arrowheads) and outside the field of view (open white arrowhead). (E–J) Manipulation of tomographic slice tilt angles revealed the shape and full extent of individual membrane invaginations from (C) and (D). (K–Q) 3D models generated from individual events (E–J) and of the entire reconstructed tomographic volume (Q). Events (red) were frequently observed close to membrane-proximal vesicles (green). Vesicles of large diameter (Ø > 60 nm; gray spheres) can be found within the vicinity of endocytic events and may represent GVs or endocytosed vesicles. (R–V) Further examples of endocytic events in stimulated MFBs. (W) Number of putative endocytic profiles per tomogram. (X) Relationship between pore diameter (P) and invagination depth (I) according to the ratio of maximum diameter (D) and pore diameter (D/p ratio = 1, light gray; D/p = > 1:1.5, dark gray; D/p = 1.5:2, blue; D/p = >2, red; respective mean values indicated by dashed lines). (Y) Parameters (D, maximum diameter; I, invagination depth; P, pore diameter) pooled from endocytic events with D/P ratios of 1.5:2 (blue) and >2 (red) for pore diameters < 100 nm. (Z) Calculated surface area of predicted endocytosed vesicles from profiles with D/P ratios >1.5. Scale bars: (A–D and R–V), 1 μm; (E–P), 100 nm. Error bars indicate mean ± SEM; ^∗∗∗^p < 0.001; ^∗∗∗∗^p < 0.0001. NS, one culture, one slice, n = 10 tomograms; ST, two cultures, two slices, n = 19 tomograms; S, two cultures, two slices, n = 19 tomograms. See also Table S1F, Figure S6, and Video S1.

One caveat of the 2D analysis is that the precise dimensions of individual events cannot be measured. This especially affects the categorization of structures with a non-uniform size distribution, because it is impossible to determine whether a given structure has been imaged at its largest size. We therefore acquired low-magnification tilt-series from 350-nm-thick plastic sections and analyzed all putative endocytic events in electron tomographic volume reconstructions (Figure 7; Video S1; Table S1F). In comparison to control conditions (Figures 7A and 7B), we found again a robust increase in the prevalence of membrane invaginations in boutons after stimulation (Figures 7C–7W). We next measured the widest diameter (D) and pore diameter (P) for each event (Figure 7X). Events with a midline outside the reconstructed volume were excluded from the analysis. This exclusion criterion explains the apparently reduced number of extremely large bulk-like events in comparison to the 2D analysis (Figure 6R). We found that all events with a D/P ratio > 1.5 and a pore size < 100 nm exhibited a relatively uniform pore diameter (46.03 ± 3.88 nm), and a comparable diameter (89.43 ± 7.78 nm) and invagination depth (87.93 ± 8.34 nm; Figure 7Y), indicating that those events are likely close to budding off from the membrane. The distribution of membrane surface areas calculated from individual invaginations (Figure 7Z) ranges between one and 11 SVs (based on a mean docked SV diameter of 46 nm). Further, larger vesicles were regularly found in the vicinity of putative endocytic structures (not quantified; Figures 7M, 7O, and 7Q) raising the interesting possibility that endocytosis preferentially occurs at distinct membrane sites or “zones” in hMFBs.

Video S1 Low-Magnification Electron Tomogram of a Stimulated Hippocampal Mossy Fiber Bouton in a Dock10-Cre;Ai32 Organotypic Slices, Related to Figure 7

In sum, our experiments indicate that hMFBs likely utilize a form of clathrin-independent compensatory membrane-retrieval in response to strong stimulation.

### Reliability of the Method under Varying Experimental Conditions

Next, we tested the robustness of our experimental system and repeated our analyses using the Nex-Cre line to drive ChR2 expression in all glutamatergic neurons in the hippocampus (Figure S2). Electrophysiological analyses of cultured slices from Nex-Cre;Ai32 animals showed that GCs reliably generated AP firing over a wide range of light intensities and with 2 and 5 ms LPs (Figure S4). To reduce the potential risk of Ca^2+^ influx through ChR2 into the terminal and double AP firing at high light intensities, we decided to work with the shorter 2 ms LP duration for experiments using this mouse line.

First, we applied a milder stimulation paradigm (20 LPs at 10 Hz, 2 mM Ca^2+^; Figures S5A–S5K) designed with the idea to induce peak short-term facilitation of responses as determined from fiber stimulation experiments at hMF synapses (Evstratova et al., 2014). Analyzing 2D electron micrographs (Table S1G), we observed a small but not significant tendency toward a reduced number of vesicles at 0–5 nm from the AZ after stimulation (Figures S5A and S5D), but the number of vesicles within 10 nm remained unchanged (Figures S5B and S5E). Moreover, no major changes in the organization of SVs within 0–100 nm were observed by 3D electron tomography (Figures S5G–S5K; Table S1H). However, in line with our findings for the Dock10-Cre;Ai32 line, stronger stimulation (100 LPs at 20 Hz, 4 mM Ca^2+^) induced a depletion of vesicles within 0–5 nm and 0–10 nm from the AZ, as assessed by 2D EM (Figures S5L–S5Q; Table S1I), and a tendency toward reduced docked SV numbers as assessed by electron tomography (Figures S5R–S5V; Table S1J). In this condition, we observed again an increase in the number of docked GVs at MF AZs, which caused an increase in the mean docked vesicle diameter, both in the 2D (Figure S5Q) and 3D analysis (Figure S5U). The observed changes in vesicle docking in Nex-Cre;Ai32 slices (4 mM Ca^2+^, 2 ms LPs) were stronger than seen using the same protocol (100 LPs at 20 Hz) in slices from the Dock10-Cre;Ai32 line (2 mM Ca^2+^, 5 ms LPs; Figure 5). This finding parallels our functional data demonstrating enhanced exocytosis at higher Ca^2+^-concentrations at GC-CA3 pyramidal cell synapses (Figure 3B–3E).

Lastly, we found that after mild stimulation, the majority of captured endocytic structures were small and located predominantly at the peri-AZ (Figures S6A–S6C; Table S1K), whereas after strong stimulation, membrane-invaginations exhibited again a range of shapes and sizes and were found both at the peri-AZ and more distally (Figures S6D–S6F; Table S1L). Electron tomographic analysis of endocytic events after strong stimulation in the presence of 4 mM Ca^2+^ (Figures S6G–S6M; Table S1M) indicated that the number of endocytic pits per tomogram and the mean surface area per putative retrieval event (Figures S6N and S6Q) were on average larger than in the presence of 2 mM Ca^2+^ using Dock10-Cre;Ai32 slices (Figures 7W and 7Z). These results are in line with the observation that compensatory clathrin-independent endocytosis in synapses of cultured hippocampal neurons scales with exocytosis (Watanabe et al., 2013b).

In summary, our results show that our method provides highly reproducible results between experiments and is compatible with the use of different mouse Cre-lines to drive ChR2 expression. Our data are in line with functional data indicating that at the hMF synapse only relatively few vesicles fuse at individual AZs in response to short AP-trains (Vyleta and Jonas, 2014) as compared to the size of the entire functional RRP as measured by long depolarizations (Hallermann et al., 2003; Midorikawa and Sakaba, 2017) and to the number of docked and membrane-proximal vesicles per AZ (Maus et al., 2020; Rollenhagen et al., 2007).

## Discussion

Key questions in modern synapse biology are how synaptic strength is dynamically regulated during synaptic plasticity processes and how exocytosis-endocytosis coupling is molecularly controlled under physiological and pathophysiological conditions. Addressing these questions requires new functional imaging approaches that can directly link defined presynaptic activity states with the organization of vesicle pools at AZs and within the entire synaptic terminal. Corresponding methodologies must offer high temporal precision, to resolve synaptic trafficking events down to the millisecond timescale, and excellent spatial resolution, to dissect the relationships between vesicles and the presynaptic AZ membrane and the location of membrane recycling events with respect to vesicle fusion sites.

“Flash-and-freeze” EM, which allows coupling of optogenetic stimulation of neurons with rapid HPF cryo-fixation for EM (Watanabe et al., 2013a, 2013b) and has become commercially available and thus accessible for the scientific community, provides an extremely powerful functional imaging solution to this problem, circumventing the otherwise static nature of EM. Indeed, several recent studies demonstrated the power of this approach in the context of neuromuscular synapses in *C. elegans* (Kittelmann et al., 2013; Watanabe et al., 2013a) and synapses of cultured mouse hippocampal neurons (Chang et al., 2018; Watanabe et al., 2013b, 2014, 2018)—but very few additional groups have so far been able to successfully implement the technology. One major obstacle preventing a routine use of the technique in the field of synapse biology has been the fact that it has so far been impossible to reliably adapt the methodology for the study of distinct synapse types embedded in brain tissue. The reason for this is that acute brain sections require supplementation of the freezing medium with external cryoprotectants (Borges-Merjane et al., 2020; Studer et al., 2014), which are not compatible with many of the functional control experiments required to directly correlate structural and functional data.

The methodology described here solves this conundrum. We developed and validated a methodological approach that combines mouse genetics, roller-tube organotypic slice cultures, electrophysiology, and “flash-and-freeze” sample cryofixation combined with EM and tomography on plastic sections, to obtain functional (e.g., electrophysiological) and ultrastructural data under the same experimental conditions.

### The Organotypic Slice Culture System

Our work represents a highly non-trivial advance that had long been unachievable due to the technical challenges and intricacies posed by cryo-fixation EM approaches (Dubochet, 1995). Our proof-of-concept experiments highlight the general applicability of our technique for the ultrastructural characterization of synapse-specific and activity-dependent membrane-trafficking processes in identified neuronal circuits, which can now be extended to other brain regions (Gähwiler, 1984; Gähwiler et al., 1997). Although results obtained in organotypic slices cannot be related one-to-one with synaptic function *in vivo*, there are multiple key advantages to this versatile preparation (Gähwiler et al., 1997) over acute brain slices (Studer et al., 2014) for cell biological studies that focus on correlating synapse function and structure at the nanoscale using “flash-and-freeze” EM:

(1) Slice recovery during the culture period permits the analysis of synapses close to the tissue surface (<10 μm; discussed in Studer et al., 2014), which is typically excellently cryopreserved in this preparation but usually most affected by acute tissue damage in acute slices.
(2) As a consequence, slice cultures of the roller-tube type can be frozen in ACSF alone without the addition of external cryoprotectants.
(3) The use of organotypic slice cultures reduces potential deleterious consequences of different slice preparation protocols (i.e., temperature transitions) that are known to affect neuronal morphology (Eguchi et al., 2020; Fiala et al., 2003).
(4) Imaged synapses in cultured slices are guaranteed to still be axonally connected to their cell bodies, which is likely important for optogenetic stimulation experiments.
(5) The slice culture approach is compatible with the use of constitutive mouse mutants with severely compromised or even perinatally lethal phenotypes (Imig et al., 2014; Siksou et al., 2009).
(6) Organotypic slices provide excellent flexibility with regard to genetic manipulations during the culture period (e.g., rescue of knockout phenotypes).
(7) Slice cultures are compatible with functional imaging technologies to study short-term and long-term synaptic plasticity processes (Helassa et al., 2018; Nägerl et al., 2008; Wiegert and Oertner, 2013).

Although we show here that basic structural (Figures S1 and S2) and functional (Figure S3) properties of hMFBs are preserved in cultured slices, circuit re-wiring and accompanying homeostatic changes during the culture period need to be acknowledged as potential limitations of the organotypic culture system. It is therefore critical that ultrastructural analyses in flash-and-freeze experiments are accompanied by corresponding functional and morphological experiments for specific stimulation paradigms.

### Caveats of the “Flash-and-Freeze” Technology

Several general experimental constraints and caveats of the “flash-and-freeze” method have to be considered when designing experiments and interpreting results in the context of synapse function:

(1) Functional EM is not a live imaging approach. Analyses are performed on static snapshots of synaptic profiles, and conclusions are drawn from probing statistically relevant changes between the analyzed conditions in the absence of a direct functional readout that corresponds to the imaged synapse and its activation status. In this regard, the highly versatile slice culture system allows for slices from the same culture to be processed in parallel but under different conditions, thereby minimizing variability.
(2) Only full-field sample illumination is possible in commercially available HPF devices. This is problematic in the context of ChR variants that are expressed ubiquitously throughout the plasma membrane and not exclusively sequestered in the soma or axon. Although synaptic transmission is efficiently blocked by the application of TTX in response to single LPs (Figure 3I), we cannot exclude the possibility that ChR2-mediated Ca^2+^-influx into the terminal alters the organization of vesicles at the membrane, especially during long stimulation paradigms (Figure 3K). Control stimulation experiments in the presence of TTX are therefore uncircumventable.
(3) While modern HPF devices can control the time interval between light stimulation and freezing with millisecond precision, biological variability ultimately limits the temporal resolution of the approach at the single synapse level. For example, light-evoked AP generation latencies are inversely related to stimulus intensity (Figures 2M and S4E) and therefore partially dependent on the homogeneity of illumination and relative sample position within the field of illumination. Moreover, structures deeper within the slice will freeze later than those close to the sapphire surface, which has a relatively high thermal conductance in comparison to the freezing medium or the tissue (Watanabe et al., 2013b). Factors that influence these parameters have to be taken into consideration. In this regard, the direct culture of tissue on sapphire discs promotes rapid heat transfer during vitrification and permits more accurate estimations of light intensities by excluding slice movements. Limiting analyses to synapses within a 10 μm distance from the sapphire disc is likely critically important for exploiting the maximal temporal resolution achievable with the method.

### Ultrastructural Correlates of Functional Synapse States

We demonstrate the power of our approach for the study of stimulus-evoked ultrastructural changes in a defined synapse type, the hMF synapse, and detected only a subtle depletion of docked SVs at hMFBs—even in response to strong stimulation protocols (100 LPs at 20 Hz). This finding is interesting as it confirms that only a sub-pool of vesicles with a higher P_r_ than the average P_r_ of the total RRP (∼500–1,400) that can be depleted by long step depolarizations (Hallermann et al., 2003; Midorikawa and Sakaba, 2017) fuses in response to AP firing during high-frequency trains (Vyleta and Jonas, 2014). Our data therefore support the notion that P_r_ is not uniformly distributed among fusion-competent vesicles, but rather that only a subset of docked, and likely molecularly primed (Imig et al., 2014; Siksou et al., 2009), SVs with a high P_r_ drive AP-evoked release (reviewed in Neher, 2015). The fact that we only saw small changes in vesicle docking after mild stimulation (20 LPs at 10 Hz) is not unexpected, in view of the well-characterized functional properties of hMFBs (Lawrence et al., 2004; Vyleta and Jonas, 2014). In an entire rat hMFB, only very few vesicles (8–9) fuse in response to a single AP and ∼53 SVs are thought to comprise the high P_r_ sub-pool of the RRP (Vyleta and Jonas, 2014). Such subtle ultrastructural changes would be virtually undetectable using 2D EM sampling approaches given that hMFBs form ∼30 AZs (Rollenhagen et al., 2007) and that only a single section and not entire AZs are sampled.

As regards synaptic endocytosis, our ultrastructural data indicate that hMFBs predominantly employ a clathrin-independent form of endocytosis directly at the end of a high-frequency stimulation train. Endocytic intermediates were captured at the peri-AZ already within the first 2 s of repetitive stimulation trains, and the number of events per hMFB, their distance from AZs, and their size increased with the amount of exocytosis. Our findings are particularly relevant in the context of functional studies that characterized endocytic membrane-retrieval in other large and structurally complex synapses, including cerebellar MFBs and the Calyx of Held synapse (Delvendahl et al., 2016; Sun et al., 2002; Wu and Wu, 2007), using presynaptic capacitance recordings:

(1) Fast, clathrin-independent endocytosis was shown to operate in cerebellar MFBs after single APs and during short trains of APs (20 APs at 300 Hz), and the kinetics of endocytosis slows down with repetitive stimulation (Delvendahl et al., 2016). Similar observations describing a stimulus-dependence of the rate of endocytosis were reported for the Calyx of Held synapse (Sun et al., 2002). Our “flash-and-freeze” EM approach allowed us to map the location of putative endocytic events in hMFBs with unprecedented spatial resolution. We show that endocytosis is restricted to the peri-AZ region after mild stimulation, whereas strong stimulation causes endocytic events also at greater distances from vesicle fusion and Ca^2+^-entry sites.
(2) We report that bulk-like endocytic events in hMFBs can be observed directly at the end of a 5 s stimulation protocol (100 stimuli at 20 Hz). This finding confirms functional observations made in the Calyx-of-Held synapse, indicating that large membrane capacitance jumps that might correspond to bulk endocytic events (de Lange et al., 2003) are detectable already within seconds after high-frequency stimulation (Wu and Wu, 2007).

Throughout our study, we observed omega-profiles at the AZ membrane directly opposing the postsynaptic density (PSD; Figure S7). Although some of these events have dimensions that are indicative of full-collapse SV fusion with the plasma membrane (Figures S7B, S7D, and S7I), many structures we captured are too large for SVs. Whether these reflect GV fusion, dense-core vesicle fusion and degranulation, or an AZ-intrinsic endocytic process, such as “kiss-and-run” triggered during sustained activity, remains to be determined. In essence, these data indicate that our experimental approach might allow the detection of exocytotic intermediates at the AZ, but a systematic optimization of incremental freezing delays is needed to increase the reliability and frequency with which such events can be captured and to assess their identity.

### Comparison with Other “Flash-and-Freeze” Studies

Previous “flash-and-freeze” EM analyses demonstrated that the number of docked SVs in synapses of cultured hippocampal neurons decreases already after a single 10 ms LP, indicating that docked SVs are indeed the first to fuse in response to an AP (Chang et al., 2018; Watanabe et al., 2013b). Further, clathrin-independent endocytosis persisted during trains of three to 100 LPs, although this mode was less predominant and/or more difficult to capture at later time points in a train than after single APs (Watanabe et al., 2014). In contrast to hMFBs, cultured neurons typically exhibit a high initial P_r_, and “flash-and-freeze” EM experiments in the corresponding studies were performed at elevated extracellular Ca^2+^ (4 mM) to further increase the likelihood of vesicle fusion. It is therefore likely that in tonic synapses, such as hMFBs, clathrin-independent, peri-AZ endocytosis remains operational even during high-frequency stimulation trains, whereas in phasic synapses, e.g., of cultured hippocampal neurons, the majority of clathrin-independent events mainly occur after the first few APs to compensate for the initially high number of fusing vesicles (Watanabe et al., 2013b, 2014).

A recent study employed “flash-and-freeze” EM to study the number of docked SVs in stimulated hMFBs in acute and organotypic interface slices and found a very strong depletion in the number of docked SVs already after a few stimuli (1–5 stimuli at 20-Hz, 2-mM Ca^2+^; Borges-Merjane et al., 2020). Further, an increase in large (∼63 nm diameter) vesicles was reported exclusively after very few LPs (five at 20 Hz), but no direct structural evidence of endocytosis (i.e., presynaptic membrane invaginations) was presented, not even upon strong stimulation (100 LPs at 20 Hz; Borges-Merjane et al., 2020). These data are difficult to reconcile with the morphological results we obtained and with the well-described functional properties of hMFBs (Lawrence et al., 2004; Vyleta and Jonas, 2014). A possible explanation for this discrepancy is that EM experiments in the corresponding study required the presence of external cryoprotectants, i.e., 15% PVP (acute slices) and 15% BSA (organotypic slices), whereas almost all flanking electrophysiological validation experiments were performed in ACSF alone (Borges-Merjane et al., 2020).

### Outlook

Our new methodological approach will make it possible to systematically dissect structural manifestations of—and contributions to—distinct functional synaptic activity and plasticity states and to determine the molecular mechanisms underlying these processes. This can be immediately achieved, for example, using shorter and more refined trains of LPs and by incrementally changing the time point of freezing after stimulation. Ultimately, our method can be combined with essentially all relevant pharmacological and genetic manipulation strategies. The latter may even include alternative optogenetic tools to study the impact of distinct intracellular signaling cascades on various cell biological processes (Oldani et al., 2020; Steuer Costa et al., 2017). In the longer term, its combination with defined genetic perturbations and its complementation by, for instance, data derived from localized proteomics or *in situ* cryo-EM will boost attempts to diagnose and predict synapse types and synapse features *in situ*, thereby promoting our understanding of neurological and psychiatric synaptopathies (Brose et al., 2019).

## STAR★Methods

### Key Resources Table

**Table:** 

REAGENT or RESOURCE	SOURCE	IDENTIFIER
**Antibodies**		

Goat anti-guinea-pig IgG Secondary Antibody, Alexa 555	Thermo Fisher Scientific	Cat# A-21435; RRID: AB_2535856
Goat anti-mouse IgG Secondary Antibody, Cy5	Thermo Fisher Scientific	Cat# A-10524; RRID: AB_2534033
Goat anti-mouse IgG Secondary Antibody, Alexa 555	Thermo Fisher Scientific	Cat# A21424; RRID: AB_141780
Goat anti-rabbit IgG Secondary Antibody, Alexa 555	Thermo Fisher Scientific	Cat# A21429; RRID: AB_2535851
Goat anti-rabbit IgG Secondary Antibody, Alexa Fluor 488	Thermo Fisher Scientific	Cat# A-11008; RRID: AB_143165
Guinea-pig polyclonal anti-Cre recombinase Antibody	Synaptic Systems	Cat# 257 004; RRID: AB_2782969
Mouse monoclonal anti-Calretinin Antibody	Swant	Cat# 6B3; RRID: AB_10000320
Mouse monoclonal anti-GFAP Antibody	Synaptic Systems	Cat# 173 111; RRID: AB_10640333
Mouse monoclonal anti-Parvalbumin Antibody	Swant	Cat# PV-235; RRID: AB_10000343
Mouse monoclonal anti-Prox1 Antibody	Thermo Fisher Scientific	Cat# P21936; RRID: AB_2539823
Rabbit polyclonal anti-GFP Antibody	MBL	Cat# MBL-598; RRID: AB_591819
Rabbit polyclonal anti-IBA1 Antibody	Synaptic Systems	Cat# 234 003; RRID: AB_10641962
Rabbit polyclonal anti-Synaptoporin Antibody	Synaptic Systems	Cat# 102 003; RRID: AB_2619748

**Chemicals, Peptides, and Recombinant Proteins**		

2,4,6-Tris(dimethylaminomethyl)phenol (DMP-30)	Electron microscopy sciences	Cat# 13600
2,3-Dioxo-6-nitro-1,2,3,4-tetrahydrobenzo[*f*]quinoxaline-7-sulfonamide disodium salt (NBQX)	HelloBio	Cat# HB0443
2-Dodecenylsuccinic acid anhydride (DDSA)	Serva	Cat# 20755.02
5-fluoro-2′-deoxyuridine	Sigma Aldrich	Cat# F0503
Acetone	Electron microscopy sciences	Cat# 10015
Alexa Fluor 555 streptavidin conjugate	Thermo Fisher Scientific	Cat# S32355; RRID: AB_2571525
Basal Medium Eagle (BME)	Thermo Fisher Scientific	Cat# 41010026
Biocytin hydrochloride	Sigma-Aldrich	Cat# B1758
Chicken Plasma	Sigma Aldrich	Cat# P3266
Cytosine β-D-arabinofuranoside hydrochlorine	Sigma Aldrich	Cat# C6645
D-APV: D-(-)-2-Amino-5-phosphonopentanoic acid	Tocris Bioscience	Cat# 0106
DAPI: 4′,6-Diamidine-2′-phenylindole dihydrochloride	Sigma-Aldrich/Roche	Cat# 10236276001
Di-Sodium hydrogen phosphate dihydrate	Merck	Cat# 1.06580.1000
Gelatin from cold water fish skin	Sigma Aldrich	Cat# G7041
Gey’s Balanced Salt Solution (GBSS)	Sigma Aldrich	Cat# G9779
GlutaMAX Supplement	Thermo Fisher Scientific	Cat# 35050061
Glycidether 100	Serva	Cat# 21045.02
Goat Serum	GIBCO	Cat# 16210-072
Hank’s Balanced Salt Solution, Ca^2+^, Mg^2+^ (HBSS)	Thermo Fisher Scientific	Cat# 24020091
Horse serum	GIBCO	Cat# 16050122
Kynurenic acid	Sigma-Aldrich	Cat# K3375
Lead (II) Nitrate	Merck	Cat# 1.07398.0100
Methylnadic anhydride (MNA)	Serva	Cat# 29452.02
Mounting glue: Aqua-Poly/Mount	Polysciences	Cat# 18606-20
Osmium tetroxide	Electron microscopy sciences	Cat# 19132
Paraformaldehyde (PFA)	Serva	Cat# 31628.02
Picrotoxin	Tocris Bioscience	Cat# 1128
Poly-L-lysine	Sigma Aldrich	Cat# P8929
Protein A (ProtA) coupled to 10 / 15 nm gold particles	Cell Microscopy Core Products, University Medical Center Utrecht, the Netherlands	N/A
Sodium citrate dihydrate	Calbiochem	Cat# 567446
Sodium dihydrogen phosphate monohydrate	Merck	Cat# 1.06346.0500
Tannic Acid 0.1%	Sigma Aldrich	Cat# 403040-100G
Tetrodotoxin	Tocris Bioscience	Cat# 1078
Thrombin from bovine plasma	Sigma Aldrich	Cat# 1.12374
Tissue-Tek O.C.T. Compound	Sakura	Cat# 4583
Triton X-100	Roche	Cat# 10789704001
Uranyl Acetate	SPI Supplies	Cat# 2624
Uridine	Sigma Aldrich	Cat# U3750

**Experimental Models: Organisms/Strains**		

Mouse: Tg(Dock10-Cre)#Stl	Kohara et al., 2014	RRID: MGI:6117432
Mouse: Nex-Cre (Neurod6^tm1(cre)Kan^)	Goebbels et al., 2006	RRID: MGI:2668659
Mouse: Ai32 (Gt(ROSA)26Sor^tm32(CAG-COP4∗H134R/EYFP)Hze^)	Madisen et al., 2012	RRID: MGI:5013789

**Software and Algorithms**		

Diffraction PSF 3D plugin	Bob Dougherty	https://imagej.net/Diffraction_PSF_3D
Fiji	Schindelin et al., 2012	https://fiji.sc; RRID:SCR_002285
GraphPad Prism 8	GraphPad Software	https://www.graphpad.com; RRID: SCR_002798
IgorPro 6.3.7.2	Wavemetrics	https://www.wavemetrics.com; RRID: SCR_000325
ImageJ	National Institutes of Health	https://imagej.nih.gov/ij; RRID: SCR_003070
IMOD software	Kremer et al., 1996	https://bio3d.colorado.edu/imod/; RRID: SCR_003297
Interactive Deconcolve 3D plugin	Bob Dougherty	https://imagej.net/Iterative_Deconvolve_3D; RRID: SCR_016246
iTEM software (Version 5.1)	Olympus Soft Imaging Solutions GmbH	N/A
Leica LAS AF	Leica Microsystems	https://www.leica-microsystems.com; RRID: SCR_013673
Patch Master v2x53/ Pulse v8.80	HEKA / Harvard Bioscience	https://www.heka.com; RRID: SCR_000034
SerialEM software	University of Colorado, Boulder, Colorado, US; Mastronarde, 2005	https://bio3d.colorado.edu/SerialEM/; RRID:SCR_017293

**Other**		

Cryostat	Leica	RRID:SCR_016844
EPC 10 double patch clamp amplifier	HEKA Elektronik	RRID:SCR_018399
JEM 2100 (200 kV)	Jeol	N/A
LED ENGIN LZ series λ 460 nm	Leica	N/A
LED, fiber-coupled KSL2/ KSL 70	Rapp Optoelectronic	N/A
Leica EM ACE600	Leica	N/A
Leica EM AFS2	Leica	N/A
Leica EM ICE	Leica	N/A
Leica EM TRIM2	Leica	N/A
Leica EM UC7 Ultramicrotome	Leica	RRID:SCR_016694
Leo912 (80 kV)	Zeiss	N/A
Orius SC1000 camera	Gatan	N/A
TCS-SP5 Confocal Microscope	Leica	N/A
Tissue chopper	McIlwain	RRID:SCR_015798
Sharp:eye CCD camera	Tröndle, TRS	N/A

### Resource Availability

#### Lead Contact

Further information and request for resources and reagents should be directed to and will be fulfilled by the Lead Contact, Benjamin H. Cooper (cooper@em.mpg.de).

#### Materials Availability

This study did not generate new unique reagents.

#### Data and Code Availability

Any raw data supporting the current study is available from the Lead Contact upon reasonable request. The study did not generate any code.

### Experimental Model and Subject Details

#### Mouse breeding

Mouse breeding and all experimental procedures used in this study were carried out with permission of the Niedersächsisches Landesamt für Verbraucherschutz und Lebensmittelsicherheit. All animals were kept according to the European Union Directive 63/2010/EU and ETS 123. Mice were housed in individually ventilated cages (type II superlong, 435 cm^2^ floor area; TECHNIPLAST) under a 12 h light/dark cycle at 21 ± 1°C with food and water *ad libitum*. The health status of the animals was checked regularly by animal care technicians and a veterinarian. In order to optogenetically stimulate subpopulations of hippocampal neurons using Cre-recombinase-dependent channelrhodopsin2 (ChR2) expression, two different cell-type specific Cre-expressing mouse lines, dentate gyrus granule cell-specific transgenic Dock10-Cre (Kohara et al., 2014) and glutamatergic forebrain neuron-specific Nex-Cre knock-in mice (Goebbels et al., 2006), were crossed into a conditional channelrhodopsin2(H134R)-enhanced yellow fluorescent protein (EYFP) expressing *Rosa26* knock-in mouse line (Ai32) (Madisen et al., 2012). All organotypic hippocampal slice cultures used in the study were prepared from mice between postnatal days 3-6 and experiments were performed between 3-5 weeks *in vitro*. For both mouse lines (Nex-Cre;Ai32 and Dock10-Cre;Ai32) mice of both genders and only Ai32/Ai32 homozygous mice were used for experiments to achieve high expression levels of the ChR2-EYFP fusion protein.

### Method Details

#### Coverslip and sapphire disc preparation

Glass coverslips (12 × 24 mm, thickness 1.5) were washed extensively with ethanol, air-died and then baked for 3 h at 200°C. For the coating procedure, coverslips were transferred with forceps into a custom-built holder, coated on both sides for 20-30 min in 0.01% poly-L-lysine (Sigma-Aldrich; #P8920) in distilled water, washed subsequently for 20 min in water, air-dried and stored at room temperature until use.

Sapphire discs (Leica; #16770158) were washed and sonicated in water and then again in pure ethanol. Individual discs were subsequently dried with dry nitrogen gas and placed into a custom-built metal holder. In preparation for the deposition of a thin (approximately 4 nm) carbon-coordinate system, each disc was covered with a SEMF2 finder grid (SPI; #2305C-XA) and the metal holder containing the grids was placed into an EM ACE600 coater (Leica). Carbon-coated discs were baked for 12h at 120°C, and then stored at room temperature until use. On the culture day, discs were briefly sterilized by UV-exposure in a tissue culture hood and then coated for 20 min with a small drop of 0.01% poly-L-lysine (Sigma-Aldrich; #P8920) in distilled water, followed by three washes with water. Each sapphire disc was then placed onto a separate glass coverslip.

#### Tissue culture

Hippocampal organotypic slice cultures were prepared using a modified roller tube method, developed by Beat H. Gähwiler (Gähwiler, 1981, 1984). Briefly, mouse pups were sacrificed on postnatal day 3-6 and the brains were removed and immediately transferred into ice-cold dissection medium [Gey’s Balanced Salt Solution (GBSS; Sigma-Adrich; #G9779) supplemented with ∼6.5 mg/ml glucose and 1 mM kynurenic acid (Sigma-Aldrich; #K3375); pH adjusted to ∼7.2 with 1M hydrochloric acid]. Hippocampi and attached entorhinal cortices were dissected from the brain with sharpened spatulas and transferred onto a tissue chopper platform of a McIlwain Tissue Chopper. Hippocampal slices (350 μm thick) were cut perpendicular to the longitudinal axis of the hippocampus and were washed off the plastic disc of the tissue chopper stage with dissection medium. Material from several animals from the same litter was pooled to increase the yield of healthy slices in a given experiment. Slices were incubated in dissection medium for 30 min at 4°C to wash out proteolytic enzymes from injured cells (Egert et al., 1998).

Afterward, each slice was transferred with a spatula into a 15 μl drop of chicken plasma (Sigma-Aldrich; #P3266) that was pipetted directly onto a sapphire disc. Immediately afterward, the chicken plasma was carefully mixed with 15 μl of ∼200 U/ml thrombin solution (Sigma-Aldrich; #1.12374) in GBSS supplemented with glucose to initiate coagulation of the plasma for the formation of a stable plasma clot. During this step, the plasma was handled with spatulas to spread the liquid from the sapphire disc onto the surrounding coverslip, which allowed them to firmly attach to the glass, and the slice was aligned to the center of the sapphire disc. Slices intended for electrophysiological experiments were attached directly to glass coverslips in 10 μl chicken plasma and 10 μl thrombin solution. Hippocampal slices were kept at room temperature for another 10-30 min (variations between different lots of chicken plasma were observed throughout this study) to allow for the plasma clot to form. Coverslips were then transferred with sterile forceps into separate plastic cell culture tubes (16 × 110 mm, Nunc; #156758).

Hippocampal slice explants were cultured at 35°C in 750 μl culture medium per tube [50 mL Basal Medium Eagle (BME; GIBCO; #41010026), 25 mL Hanks’ Balanced Salt Solution (HBSS; GIBCO; #24020091), 25 mL Horse Serum (GIBCO; #16050122; heat-inactivated for 30 min at 56°C), 1 mL GlutaMAX (GIBCO; #35050061), and supplemented with ∼650 mg/100 mL glucose]. The tubes were placed into a Heraeus Incudrive-S incubator containing a motor-driven roller drum (10 rotations per hour) which was tilted by ∼7°C with respect to the horizontal axis. Slices were treated with antimitotics [∼4 μM 5-fluoro-2′-deoxyuridine (Sigma-Aldrich; #F0503), cytosine β-D-arabinofuranoside hydrochlorine (Sigma-Aldrich; #C6645), and uridine (Sigma-Aldrich; #U3750)] for ∼16-24 h on days *in vitro* (DIV) 2-3. Medium was subsequently changed every 5-7 days. Slices thinned out during the culture period to a few cell layers (<150 μm).

Hippocampal explants were cultured in the dark, however slices were occasionally exposed to light, for example during the dissection procedure, medium changes and occasional quality control at the cell culture microscope.

#### Electrophysiology

Electrophysiological experiments were performed on organotypic slices from Dock10-Cre;Ai32 and Nex-Cre;Ai32 animals between 3-5 weeks *in vitro*. Slices were placed into the recording chamber at low ambient light and allowed to recover for 20 min in the dark before recordings commenced. Neurons were visualized using near IR illumination through a 60x (Olympus, 0.9 NA) or 10x (Zeiss, 0.30 NA) objective. All recordings were done in a dark environment using the minimum light required to illuminate the instrumentation. The remaining dim ambient illumination did not activate ChR as verified by the unchanged membrane conductance in comparison to complete darkness.

Hippocampal tissue explants still attached to the glass coverslip were mounted into a recording chamber and perfused throughout the experiment with carbogenated and warmed to near physiological temperatures (∼35°C) artificial cerebrospinal fluid (ACSF) containing (in mM): 120 NaCl, 2 KCl, 1 KH_2_PO_4_, 20 D-glucose, 26 NaHCO_3_, 1 MgCl_2_ and either 2 or 4 CaCl_2_.

Patch pipettes were pulled from borosilicate glass capillaries with filament (Science Products) to have an open-tip resistance of 3-5 MΩ when filled with intracellular solution. For current clamp recordings the intracellular solution contained (in mM): 120 K-gluconate, 30 KCl, 0.2 EGTA, 2 MgCl_2_, 2 Na-ATP, 10 HEPES, 320 mOsm, pH 7.2. For whole-cell voltage-clamp recordings of EPSCs in CA1 and CA3 pyramidal cells in response to fiber or optogenetic stimulation, patch pipettes were filled with an intracellular solution containing (in mM): 130 K-gluconate, 10 KCl, 10 EGTA, 2 MgCl_2_, 2 Na-ATP, 10 HEPES, 2 QX-134; 330 mOsm, pH 7.2. Some cells were filled with intracellular solution containing 0.4% biocytin for a light microscopic characterization of the morphologies of CA3 pyramidal cells and dentate gyrus granule cells in cultured slices (Figure S2). We did not correct for the liquid junction potential.

Single APs or trains of APs were induced in ChR2(H134R)-expressing CA3 pyramidal neurons or dentate gyrus GCs with pulses of blue light (470 nm) through a 60x or 10x objective using a fiber-coupled LED (KSL 70 or KSL2, Rapp OptoElectronic).

A HEKA EPC10 amplifier was controlled by the “Pulse” software (HEKA Elektronik). Sampling intervals and filter settings were 20 μs and 5 kHz, respectively. For voltage-clamp recordings, the membrane potential was set to −65 mV.

For characterization of discharge and membrane properties of pyramidal and DG neurons in current-clamp mode, excitatory and inhibitory synaptic transmission was blocked by adding 50 μM D-(-)-2-Amino-5-phosphonopentanoic acid (D-AP5; Tocris; #0106), 2 μM 2,3-Dioxo-6-nitro-1,2,3,4-tetrahydrobenzo[*f*]quinoxaline-7-sulfonamide disodium salt (NBQX; Hello Bio; #HB0443), and 100 μM picrotoxin to the ACSF. AP-firing properties of individual neurons were tested in response to varying light pulse durations (1-10 ms) and light intensities (light power at the slice ranging from ∼1-40 mW/mm^2^). In Figures 2J and S4B traces exemplify APs and ChR2-mediated currents (I_ChR2_). For comparison, current-clamp responses to injection of small hyper- or depolarizing current pulses triggering single APs or trains of APs were acquired.

During voltage-clamp recordings, no GABA antagonists were applied, but occasionally the holding potential was adjusted to the experimentally determined reversal potential of GABAergic IPSCs to isolate EPSCs (less than ± 5 mV adjustment). For electrical stimulation, a monopolar stimulating electrode (3-5 MΩ) was filled with ACSF and placed either in the *stratum radiatum* ∼30-50 μm away from the CA1 pyramidal cell or in the *statum lucidum* in the vicinity of MFBs (∼5-10 μm away of the recorded CA3 dendrites). The position of the electrode was adjusted until excitatory postsynaptic currents (EPSCs) with a delay of <6 ms from the onset of the stimulus were measured. The stimulus strength was set to ≤7 V and pulses duration was 0.1 ms.

All offline analysis of electrophysiological data was performed in Igor Pro (Wavemetrics). Voltage-clamp recordings were leak corrected and low-pass filtered with a cut-off frequency f_c_ = 3 kHz using a 10-pole digital Bessel filter.

#### High-pressure freezing

Organotypic slices were frozen with a Leica EM ICE high pressure freezing device equipped with a heated stage and a blue LED module (LED ENGIN LZ series λ 460 nm; Leica; #16771842) that delivers, depending on variation between independent LED modules, a maximum irradiance between 5.5-8.0 mW/mm^2^ at the sapphire disc surface in the freezing chamber (Leica Microsystems Technical Data Sheet). The relative LED intensity measured within the central 2 × 2 mm region of the sapphire disc (Figure 1E, adapted with permission from Leica Microsystems) is between 80%–100% of maximum LED intensity as measured by scanning a foil aperture (Ø = 1mm), positioned approximately 0.5 mm below the upper transparent half cylinder, in 0.5 mm steps in the x and y directions above a PD300-TP photodiode sensor connected to an OPHIR Nova II power meter (experiment performed by Leica as specified in technical data sheet). We therefore estimate that neurons located within this central 2 × 2 mm area of carbon coordinate system deposited on the sapphire disc are exposed to irradiance values within the range of approximately 4.4-8.0 mW/mm^2^.

For each experiment, slices from the same culture were frozen on the same day after either light stimulation (LS), light stimulation in the presence of 1 μM tetrodotoxin (TTX; Tocris Bioscience; #1078) (LS+T), or without prior stimulation (no stimulation, NS). One day before the experiment, organotypic slices were changed to fresh culture medium and on the day of the experiment, the osmolarity of the medium in the cell culture tubes was assessed (∼330-340 mOsm).

Individual sapphire discs with the hippocampal organotypic slices attached to the surface were carefully removed from the underlying glass coverslips and transferred into carbogenated ACSF (120 mM NaCl, 2 mM KCl, 1 mM KH_2_PO_4_, 20 mM D-glucose, 26 mM NaHCO_3_, 1 mM MgCl_2_ and either 2 mM or 4 mM CaCl_2_, 50 μM D-AP5, 1 μM NBQX; osmolarity adjusted to ∼310-320 mOsm with D-glucose) in a recovery chamber inside of a water bath set to 37°C. Some slices were incubated in the presence of 1 μM TTX. Hippocampal tissue explants were allowed to recover from the transition from culture medium to ACSF for 15-20 min in the dark and in presence of the indicated receptor and channel blockers.

All further steps were performed under darkroom conditions with dim red lid supplied by a Kindermann dukalux x-tronic (type 2580; 640 nm) and a KL1500 LCD light source (Schott) equipped with a red insert filter (Schott; #562 44 287 3). Briefly, a few ml of the carbogenated 37°C ACSF were transferred with a plastic Pasteur pipette from the recovery chamber into a plastic Petri dish used for preparation of the sapphire disc freezing assembly. Using fine forceps under a stereomicroscope, an uncoated sapphire disc (“base”), a stainless steel spacer ring (6 × 5 x 0.15 mm; Engineering Office M. Wohlwend GmbH; #1258), and the sapphire disc harboring the organotypic slice (“lid”) were submerged in the pre-warmed ACSF. In this completely submerged state, the spacer ring was placed on the uncoated sapphire disc and the disc harboring the tissue explant was carefully inverted and aligned on top of the preassembled “base” to serve as the “lid,” being careful not to trap air bubbles between the discs. The entire sapphire disc assembly was then transferred with fine forceps from the Petri dish to a sample holder middle plate (Ø 6.0 mm; Leica; #16771838) and covered with a rubber cover ring (500 μm; Leica; #16771884). Excess liquid was removed with Whatman filter paper and the middle plate with the sample was positioned on top of a transparent half cylinder (Leica; #16771846) on the heated stage of the HPF device. To maintain samples at the near-physiological temperatures used for the complementary electrophysiological calibration experiments, the heated loading stage of the EM ICE was nominally set to 37 °C and the high-pressure freezing chamber was maintained at 35°C.

Slices were allowed to acclimatize for 8 s in the dark freezing chamber prior to freezing or to the initiation of the light stimulation protocol. For optogenetic stimulation of hippocampal mossy fiber synapses, slices were illuminated with a 20 Hz train of 100 light pulses of either 2 or 5 ms duration or with a 10 Hz train of 20 light pulses of 2 ms duration. Cryo-fixed samples were stored in liquid nitrogen until further processing.

#### Automated freeze substitution

Cryo-fixed samples were removed from liquid nitrogen storage and immersed in anhydrous acetone precooled to −90°C in a Leica AFS2 automated freeze substitution device. Upon separation of the sapphire disc assembly, the slice-bearing sapphire disc was carefully loaded with cryoforceps into a custom-built aluminum sapphire disc revolver (Figure 1F) immersed in 0.1% tannic acid in anhydrous acetone at −90°C. The revolver was designed to protect material frozen on sapphire discs from abrasive contact with other discs or vessel walls while enabling easy exchange of freeze substitution media.

Automated freeze substitution was performed as previously described (Imig and Cooper, 2017; Rostaing et al., 2006). Briefly, samples were incubated in 0.1% tannic acid in anhydrous acetone for 4 days at −90°C, fixed with 2% osmium tetroxide in anhydrous acetone with the temperature slowly ramping up over several days to 4°C, washed in acetone and brought to room temperature for EPON infiltration and embedding. For the embedding, sapphire discs were placed slice-upward on Parafilm-coated glass slides, gel-capsules filled with 100% EPON were inverted on the specimen, and the samples were polymerized at 60°C for 24-48 h. Polymerized blocks were removed from the glass slides and the sapphire discs were carefully trimmed off by brief contact with a liquid nitrogen-cooled cotton swab and razor blades to expose the carbon-coordinate system on the block surface. With the help of the coordinate system, samples were further trimmed down (Leica EM TRIM2) to contain the dentate gyrus, CA3 and CA1 regions of the hippocampus and to fit an EM grid in preparation for ultramicrotomy.

#### Ultramicrotomy

A Leica EM UC7 ultramicrotome was used to cut sections from EPON-embedded slices according to the following sequence spanning a cutting depth of approximately 2 μm: 4 × 200 nm, 3 × 60 nm, and 3 × 350 nm. This sequence was repeated and the last 350 nm-thick section of each sequence was collected on a glass slide and Nissl stained to assess the structural organization of the hippocampal formation. Ultrathin (60 nm) and semithin (200 nm and 350 nm) sections were collected on Formvar-filmed, carbon-coated, and glow-discharged copper 100-mesh grids for 2D transmission electron microscopy and 3D electron tomography, respectively. Ultrathin sections were contrasted with 1% aqueous uranyl acetate and 0.3% Reynold’s lead citrate, and semithin sections were treated with Protein A coupled to 10 nm or 15 nm gold particles (Cell Microscopy Center, Utrecht; the Netherlands), which served as fiducial markers for high magnification (30,000 x) and low magnification (5,000 x) tomography, respectively. The quality of the ultrastructural tissue preservation was assessed in ultrathin sections based on previously established criteria (Möbius et al., 2010), and specimen exhibiting signs of ice-crystal formation or neuronal degeneration (occasionally observed in particularly thick tissue slices in the CA1 region) were excluded from the analysis.

#### Electron tomography and data analysis

Tilt series acquisition of active zones and boutons from Schaffer collateral and mossy fiber synapses for electron tomography was performed as previously described (Imig and Cooper, 2017; Maus et al., 2020). All samples were blinded before imaging and analysis. Single-axis tilt series were acquired on a JEOL JEM-2100 200 kV transmission electron microscope from −60/-55 to +55/+60 in 1° increments and binned by a factor of two on an Orius SC1000 camera (Gatan). SerialEM (Mastronarde, 2005) was used to acquire automated tilt-series at either 5,000 x or 30,000 x magnification.

Active zones were selected for high magnification tomographic analysis on 200 nm thick sections when the synaptic cleft was parallel to the tilt axis and visible at 0° stage tilt. For mossy fiber synapses, only putative release sites contacting CA3 pyramidal cell dendritic spines in the *stratum lucidum* were selected. Final tomograms were reconstructed binned by a factor of three (1.554 nm isotropic voxel size of the final tomogram) and segmented for analysis using the IMOD package (Kremer et al., 1996). All vesicles within 100 nm of the active zone were segmented as previously described (Maus et al., 2020). The active zone was defined as the presynaptic membrane apposing the postsynaptic density (PSD). However, the full extension of the PSD was sometimes difficult to determine, therefore also the widening of the synaptic cleft and the presence of electron-dense cleft material were used as additional criteria for the segmentation of the active zone. The shortest distance between the vesicle membrane and the active zone was calculated using the mtk program of the IMOD package (Kremer et al., 1996) and the active zone area and vesicle diameters were extracted from the segmented model using the imodinfo program. Vesicles were scored “docked” when no measurable distance between the inner leaflet of the plasma membrane and the outer leaflet of the vesicle membrane could be determined. The number of vesicles in discrete bins was normalized to the measured active zone area. For mossy fiber synapses, vesicles with a diameter less than 60 nm were classified as “SVs,” whereas vesicles with a diameter exceeding 60 nm and having no prominent dense core were classified as giant vesicles (Henze et al., 2002; Maus et al., 2020). Vesicles that contained a prominent electron-dense core were considered dense-cored vesicles (DCVs). For illustrative purposes, figures depicting tomographic sub-volumes represent an overlay of seven consecutive tomographic slices. For optimal visualization of individual events, enlarged subvolumes were rotated in 3D using the slicer tool of the IMOD software (Kremer et al., 1996).

To increase the likelihood of capturing activity-dependent endocytic membrane trafficking events, larger subvolumes of mossy fiber synaptic terminals were imaged at 5,000 x magnification in 350 nm-thick sections. Final tomograms were reconstructed unbinned (2.616 nm isotropic voxel size of the final tomogram) and used to score the number of omega-shape exo- or endocytic events per mossy fiber synaptic profile. Only presynaptic membrane invaginations were classified as endocytic pits, since it was difficult to discriminate between already pinched-off endocytic vesicles and the giant vesicles that are also known to exist at rest in the terminals of this highly specialized synapse type (Henze et al., 2002; Maus et al., 2020). Only invaginations captured entirely within the tomographic subvolume were used to quantify endocytic membrane surface areas. Invaginations were segmented on each tomographic slice in 3dmod. At the presynaptic membrane, the center of positive curvature was used to define invagination boundaries with the plasma membrane and the spherical interpolation tool was used to estimate curved membrane profiles obscured by missing wedge (Mastronarde, 1997) artifacts. Segmentation contours were meshed as separate objects and corresponding surface areas were extracted using the *imodinfo* command. For illustrative purposes, figures depicting tomographic sub-volumes represent an overlay of four consecutive tomographic slices and for the enlargements of individual fusion/endocytosis events two consecutive tomographic slices. For optimal visualization and quantification of individual events, enlarged subvolumes were rotated in 3D using the slicer tool of the IMOD software (Kremer et al., 1996) to reveal their maximal extent. Fiji software (Schindelin et al., 2012) was used to quantify structural endocytic parameters (Kukulski et al., 2012), including the maximal diameter, measured as the largest distance separating opposing bilayer midlines; maximal invagination depth, as measured from the bilayer midline at the invagination tip to the estimated position of the presynaptic membrane if it spanned the pore; and minimal pore width, measured as the closest approach between opposing bilayer midlines.

#### Two-dimensional analysis

All samples were blinded before imaging and analysis and mossy fiber and Schaffer collateral synaptic profiles were chosen randomly. Electron micrographs were acquired with a sharp:eye CCD camera (Tröndle, TRS) at 20,000 x magnification and a pixel spacing of 0.592 nm using the iTEM software (Version 5.1, Olympus Soft Imaging Solutions). To capture entire profiles of mossy fiber boutons, the multiple image acquisition and alignment feature of the iTEM software was used. To minimize the number of vesicles that were quantified false-positively as “docked” due to high curvature of the presynaptic plasma membrane in the volume of the ultrathin section (Imig and Cooper, 2017), only active zones with a clearly visible (“clear-cut”) plasma membrane were included in the analysis. Since many release sites formed by mossy fiber terminals onto complex dendritic spines exhibited a high degree of curvature, only a subset of active zones in a mossy fiber profiles was usually included in the analysis. Number of vesicles in discrete bins (i.e., 0-5 nm and 0-10 nm from the active zone membrane) were normalized to the measured active zone length and reported normalized to the mean number of vesicles in the unstimulated control condition.

The morphometric analysis of endocytic invaginations captured in ultrathin sections was performed using Fiji software (Schindelin et al., 2012) and based on structural parameters (Kukulski et al., 2012), including maximal invagination depth, as measured from the bilayer midline at the invagination tip to the estimated position of the presynaptic membrane if it spanned the pore, and the minimal pore width, measured as the closest approach between opposing bilayer midlines. Note that in 2D analyses, these two parameters are subject to underestimation.

#### Light microscopic analysis

For the immunohistochemical characterization of hippocampal organotypic explants, slices on glass coverslips were fixed by overnight immersion in 4% PFA in 0.1 M PB (pH 7.4). Slices were subsequently washed in 0.1 M PB (pH 7.4) and removed from the glass coverslip using a razor blade and cryoprotected by immersion in an increasing gradient of 20% and 30% sucrose in 0.1 M PB (pH 7.4). Slices were briefly immersed in liquid Tissue-Tek® OCT compound (Sakura, #4583) and then placed flat on the base of a small freezing mold made out of aluminum foil. The tissue was then covered with liquid OCT. The OCT-filled form was then rapidly frozen on a liquid nitrogen-cooled aluminum block, the foil was removed, and the frozen OCT block was mounted on a cryostat (Leica CM3050 S) stub. Cryosections (10 μm-thick) were thaw-mounted on Superfrost slides. Slides were washed briefly in 0.1 M PB (pH 7.4) and incubated 90 min at RT in 10% normal goat serum (NGS,) 0.3% Triton X-100, and 0.1% cold-water fish skin gelatine (FSG; Sigma-Aldrich; #G7041) in 0.1 M PB (pH 7.4). Slices were then incubated overnight at 4°C in 3% NGS, 0.3% Triton X-100 and 0.1% FSG in 0.1 M PB (pH 7.4) containing primary antibodies. The primary antibodies used in the study included polyclonal rabbit anti-GFP (MBL; # MBL-598; 1:1,500 dilution), polyclonal guinea-pig anti-Cre-recombinase (Synaptic Systems; #257 004; 1:500 dilution), polyclonal rabbit anti-Synaptoporin (Synaptic Systems; #102 003; 1:500 dilution), monoclonal mouse anti-Prox1 (Thermo Fisher; #P21936; 1:800 dilution), monoclonal mouse anti-Parvalbumin (Swant; #PV-235; 1:500 dilution), monoclonal mouse anti-Calretinin (Swant; #6B3; 1:500 dilution), monoclonal mouse anti-GFAP (Synaptic Systems; #173 111; 1:500 dilution), and polyclonalclonal rabbit anti-IBA1 (Synaptic Systems; #234 003; 1:1000 dilution). Slices were subsequently washed in 0.1 M PB (pH 7.4) and primary antibodies were visualized by 2 hr incubation at RT in 5% NGS, 0.1% Triton X-100 and 0.1% FSG in 0.1 M PB (pH 7.4) containing fluorophore-coupled secondary antisera. The secondary antibodies used in the study included goat anti-rabbit Alexa 488 (Thermo Fisher; #A11008; dilution 1:1000), goat anti-rabbit Alexa 555 (Thermo Fisher; #A21429; dilution 1:1000), goat anti-mouse Alexa 555 (Thermo Fisher; #A21424; dilution 1:1000), goat anti-guinea-pig Alexa 555 (Thermo Fisher; #A21435; dilution 1:1000), and goat anti-mouse Cy5 (Thermo Fisher; #A10524; dilution 1:1000). Slices were washed in 0.1 M PB, then incubated for 30 min at RT in 300 nM DAPI (Sigma-Aldrich; #10236276001) to label cell nuclei. Following a brief wash in 0.1 M PB, slides were dipped in distilled water and Menzel-Gläser #1.5 coverslips were mounted using Aqua-Poly/Mount mounting medium (Polysciences, Inc.; #18606-20).

Light microscopic analysis of ChR2-EYFP expression in dentate gyrus granule cells of NEX-Cre;Ai32 slices was performed using a widefield microscope (Leica DMi8, Thunder 3D Live Cell Imaging System) equipped with a Spectra X light engine (Lumencor), an HC PL APO CS2 63x/1.30 GLYC UV objective, a 4.2 MP DFC9000 sCMOS camera (Leica), and LAS X software (Leica). Unbinned multichannel Z stacks (16-bit; voxel sizes x, y, 0.103 μm; z, 0.251 μm) were acquired throughout a depth of 7.53 μm to detect anti-GFP (Alexa 555 signal) and Prox-1 (Cy5 signal) immunoreactive granule cells. Identical acquisition parameters, initially optimized in Nex-Cre;Ai32 slices to ensure signal intensities were within linear range, were identically applied for imaging wild-type slices that had been labeled in parallel with the same antibody cocktails. Images from Nex-Cre;Ai32 and wild-type slices were similarly subjected to identical processing steps: Maximum projections were generated with LAS X software and FIJI software (Schindelin et al., 2012) was used for 8-bit conversion and LUT assignment.

Confocal light microscopic analysis of biocytin-filled cells was performed to i) validate that electrophysiological recordings were performed for the correct cell types and that ii) the cell morphology and neuronal connectivity was preserved in hippocampal organotypic slice cultures. Biocytin-filled CA3 pyramidal cells (see Electrophysiology section above for detailed procedure) were fixed for light microscopic analysis by overnight immersion of the coverslip-mounted slice in 4% PFA in 0.1 M PB (pH 7.4). Slices were washed in 0.1 M PB (pH 7.4) and then incubated overnight at 4°C in 10% NGS, 0.3% Triton X-100, and 0.1% FSG in 0.1 M PB (pH 7.4). Biocytin-filled cells were visualized by incubation of slices for 3 hr at RT in Steptavidin-Alexa 555 (Thermo Fisher; #S32355; 1:1000 dilution) in 5% NGS, 0.1% Triton X-100 and 0.1% FSG in 0.1 M PB (pH 7.4). Slices were washed in 0.1 M PB (pH 7.4) and cell nuclei were labeled by a 30 min incubation in 300 nM DAPI (Sigma-Aldrich; #10236276001). Following final washing steps in 0.1 M PB (pH 7.4), slices were mounted onto Superfrost glass slides using Aqua-Poly/Mount mounting medium (Polysciences, Inc., #18606-20).

Confocal laser scanning micrographs of were acquired with a Leica TCS-SP5 confocal microscope equipped with a tuneable white light laser, a resonant scanner, hybrid GaAsP detectors, and a motorized stage. Tiled images were acquired with a HCX PL APO 40x (NA = 1.25) oil immersion objective to generate overviews of immunolabelled organotypic slices (pinhole = 3.0 AU, single-plane, pixel size x, y = 0.19, 0.19 μm) and biocytin-filled cells (pinhole = 3.0 AU, multi-plane z stacks, voxel size x, y, z = 0.19, 0.19, 2.5 μm). In biocytin-filled material, high-magnification images of mossy fiber boutons and complex spines on CA3 pyramidal cells were acquired with a HCX PL APO CS 100x (NA = 1.4) oil immersion objective (pinhole = 1.0 AU, multi-plane z stacks, voxel size x, y, z = 50.6, 50.6, 125.9 nm). To better resolve their multi-compartmental morphology, complex spines were subjected to spatial deconvolution by use of two ImageJ (National Institutes of Health; Bethesda, MD) plugins: theoretical point spread functions (PSF) were generated using Diffraction PSF 3D and deconvolution was performed using Iterative Deconvolve 3D (Dougherty, 2005). For illustrative purposes complex spines were rendered in 3D using FIJI (Schindelin et al., 2012) and IMOD (Kremer et al., 1996) software packages. Briefly, the deconvolved confocal z stacks were processed to generate isotropic voxels (reslice function, FIJI), converted to MRC file format (tif2mrc, IMOD), segmented by a threshold-based algorithm (imodauto, IMOD), and rendered in 3D (3dmod, IMOD).

The relative proportions of Cre-recombinase immunoreactive cells was quantified in the dentate gyrus and CA3 hippocampal subfields using 10 μm-thick cryosections through organotypic slices from Nex-Cre;Ai32 and Dock10-Cre;Ai32 mice, respectively. A minimum of three slices from independent cultures were analyzed from Nex-Cre;Ai32 and Dock10;Ai32 lines. Tiled, single-plane confocal images encompassing entire dentate gyrus and CA3 subfields were acquired with a 40x oil-immersion objective (HCX PL APO lambda blue 40x 1.25 OIL UV; pixel x,y = 0.189 μm, pinhole = 3 AU) with gain settings selected to maintain DAPI and Cre-recombinase signals in linear range. Using FIJI software (Schindelin et al., 2012), the DAPI channel was thresholded and a mask comprising individually demarcated DAPI-labeled nuclei was generated by applying erode and watershed binary processing functions followed by the analyze particles feature (size, 30-Infinity; circularity 0.5-1.0; Display Bare Outlines). Using the nuclear masks, Cre-recombinase labeling ‘background’ was quantified by measuring either the mean Cre-recombinase signal obtained in nuclei located outside of the principal cell layers (CA3 *stratum radiatum*), or in nuclei within the CA3 *stratum pyramidale*, for Nex-Cre;Ai32 and Dock10-Cre;Ai32 slices, respectively. The threshold for ‘real’ Cre-recombinase signal was arbitrarily set at two standard deviations above mean background levels. The relative proportion of nuclei harboring above-threshold Cre-recombinase signals in a given subfield was compared to total numbers of DAPI nuclei to estimate the proportion of ChR2-expressing cells (93.7%, Nex-Cre;Ai32 CA3 *stratum pyramidale*; 91.2%, Dock10-Cre;Ai32 dentate gyrus GCs). These values likely represent underestimates since the outlined approach is insensitive to the presence of glial or inhibitory cells within the measured cell populations.

### Quantification and Statistical Analysis

Data are represented as mean ± SEM unless indicated otherwise. Statistical analyses were carried out using GraphPad Prism software 8 (^∗^ when p < 0.05; ^∗∗^ when p < 0.01, ^∗∗∗^ when p < 0.001, and ^∗∗∗∗^ when p < 0.0001). For comparisons of multiple conditions statistical significance was tested by one-way ANOVA followed by Student's t-test with Bonferroni correction as a post-test if the dataset was normally distributed determined using the Kolmogorov-Smirnov normality test. If the dataset was not normally distributed, a Kruskal-Wallis ANOVA on ranks was performed with a Dunn’s comparison of all columns as a post-test to probe for statistical significance. Unless indicated otherwise, (n) refers to the number of tomograms (3D) or active zones (2D) analyzed for each experiment or the number of cells measured for electrophysiological experiments pooled over several slices. Numbers of biological replications for each experiment are listed in the Figure legends and Table S1.

## Supplemental Information

Document S1. Figures S1–S7

Table S1. Ultrastructural Parameters, Related to Figures 4, 5, 6, 7, S1, S5, and S6 (A) 3D Electron tomographic (30,000× magnification) analysis of AZ-proximal (0-100 nm) vesicle pools at MF-CA3 pyramidal cell AZ profiles in wild-type organotypic slices frozen at 2mM and 4 mM extracellular Ca^2+^. Related to Figures S1A–S1H. (B) 3D Electron tomographic (30,000× magnification) analysis of AZ-proximal (0–100 nm) vesicle pools at Schaffer Collateral AZ profiles in wild-type organotypic slices frozen at 2mM and 4 mM extracellular Ca^2+^. Related to Figures S1I–S1O. (C) 2D TEM analysis of AZ-proximal Vesicles (0 to 10 nm) at MF-CA3 pyramidal cell AZ profiles in Dock10-Cre;Ai32 organotypic slices frozen after strong stimulation (100 × 20 Hz, 5 ms pulses, 2 mM extracellular Ca^2+^). Related to Figures 4E–4J. (D) 3D Electron tomographic (30,000× magnification) analysis of AZ-proximal (0–100 nm) vesicle pools at MF-CA3 pyramidal cell AZ profiles in Dock10-Cre;Ai32 organotypic slices frozen after strong stimulation (100x 20Hz, 5 ms pulses, 2 mM extracellular Ca^2+^). Related to Figures 5G–5N. (E) 2D TEM analysis of putative endocytic events at MF-CA3 pyramidal cell AZ profiles in Dock10-Cre;Ai32 organotypic slices frozen after strong stimulation (100 × 20 Hz, 5ms pulses, 2mM extracellular Ca^2+^). Related to Figures 6P–6S. (F) 3D Electron tomographic (5,000× magnification) analysis of putative endocytic events at MF-CA3 pyramidal cell AZ profiles in Dock10-Cre;Ai32 organotypic slices frozen after strong stimulation (100 × 20 Hz, 5 ms pulses, 2 mM extracellular Ca^2+^). Related to Figures 7W–7Z. (G) 2D TEM analysis of AZ-proximal Vesicles (0–10 nm) at MF-CA3 pyramidal cell AZ profiles in Nex-Cre;Ai32 organotypic slices frozen after mild stimulation (20 × 10 Hz, 2ms pulses, 2 mM extracellular Ca^2+^). Related to Figures S5A–S5F. (H) 3D Electron tomographic (30,000× magnification) analysis of AZ-proximal (0–100 nm) vesicle pools at MF-CA3 pyramidal cell AZ profiles in Nex-Cre;Ai32 organotypic slices frozen after mild stimulation (20 × 10Hz, 2 ms pulses, 2 mM extracellular Ca^2+^). Related to Figures S5G–S5K. (I) 2D TEM analysis of AZ-proximal Vesicles (0–10 nm) at MF-CA3 pyramidal cell AZ profiles in Nex-Cre;Ai32 organotypic slices frozen after strong stimulation (100 × 20 Hz, 2 ms pulses, 4 mM extracellular Ca^2+^). Related to Figures S5L–S5Q. (J) 3D Electron tomographic (30,000× magnification) analysis of AZ-proximal (0–100 nm) vesicle pools at MF-CA3 pyramidal cell AZ profiles in Nex-Cre;Ai32 organotypic slices frozen after strong stimulation (100 × 20 Hz, 2 ms pulses, 4 mM extracellular Ca^2+^). Related to Figures S5R–S5V. (K) 2D TEM analysis of putative endocytic events at MF-CA3 pyramidal cell AZ profiles in Nex-Cre;Ai32 organotypic slices frozen after mild stimulation (20 × 10 Hz, 2 ms pulses, 2 mM extracellular Ca^2+^). Related to Figures S6A–S6C. (L) 2D TEM analysis of putative endocytic events at MF-CA3 pyramidal cell AZ profiles in Nex-Cre;Ai32 organotypic slices frozen after strong stimulation (100 × 20 Hz, 2 ms pulses, 4 mM extracellular Ca^2+^). Related to Figures S6D–S6F. (M) 3D Electron tomographic (5,000× magnification) analysis of putative endocytic events at MF-CA3 pyramidal cell AZ profiles in Nex-Cre;Ai32 organotypic slices frozen after strong stimulation (100 × 20Hz, 2 ms pulses, 4 mM extracellular Ca^2+^). Related to Figures S6N–S6Q. AZ, active zone; D, widest diameter; GV, giant vesicle (>60 nm in diameter); I, Invagination depth; MFB, mossy fiber bouton; NS, No Stimulation; P, pore diameter; S, Stimulation; SC, Schaffer Collateral; ST, Stimulation in the presence of 1 μM TTX; SV, synaptic vesicle (<60 nm in diameter); TTX; TTX, tetrodotoxin; WT, wild-type. All values are represented as mean ± SEM. Normally distributed data sets (two conditions: Student’s unpaired t test; More than two conditions: One-way ANOVA plus Bonferroni’s multiple comparison post-test). Not normally distributed data sets (two conditions: Mann-Whitney U test; more than two conditions: Kruskal-Wallis test plus Dunn’s comparison post-test).

Video S2 Low-Magnification Electron Tomogram of a Stimulated Hippocampal Mossy Fiber Bouton in a Nex-Cre;Ai32 Organotypic Slices, Related to Figure 7

Document S2. Article plus Supplemental Information
