# Supplementary material for: Ultrastructural Imaging of Activity-Dependent Synaptic Membrane-Trafficking Events in Cultured Brain Slices
Source: Neuron. 2020 Dec 9;108(5):843–860.e8. doi: 10.1016/j.neuron.2020.09.004 (PMC7736621; doi:10.1016/j.neuron.2020.09.004)
Supplement: Document S1. Figures S1–S7 [file mmc1.pdf]

Neuron, Volume *108*

Supplemental Information

Ultrastructural Imaging of Activity-Dependent Synaptic Membrane-Trafficking Events in Cultured Brain Slices

Cordelia Imig, Francisco José López-Murcia, Lydia Maus, Inés Hojas García-Plaza, Lena Sünke Mortensen, Manuela Schwark, Valentin Schwarze, Julie Angibaud, U. Valentin Nägerl, Holger Taschenberger, Nils Brose, and Benjamin H. Cooper

Neuron, Volume *108*

Supplemental Information

Ultrastructural Imaging of Activity-Dependent Synaptic Membrane-Trafficking Events in Cultured Brain Slices

Cordelia Imig, Francisco José López-Murcia, Lydia Maus, Inés Hojas García-Plaza, Lena Sünke Mortensen, Manuela Schwark, Valentin Schwarze, Julie Angibaud, U. Valentin Nägerl, Holger Taschenberger, Nils Brose, and Benjamin H. Cooper

Figure S1

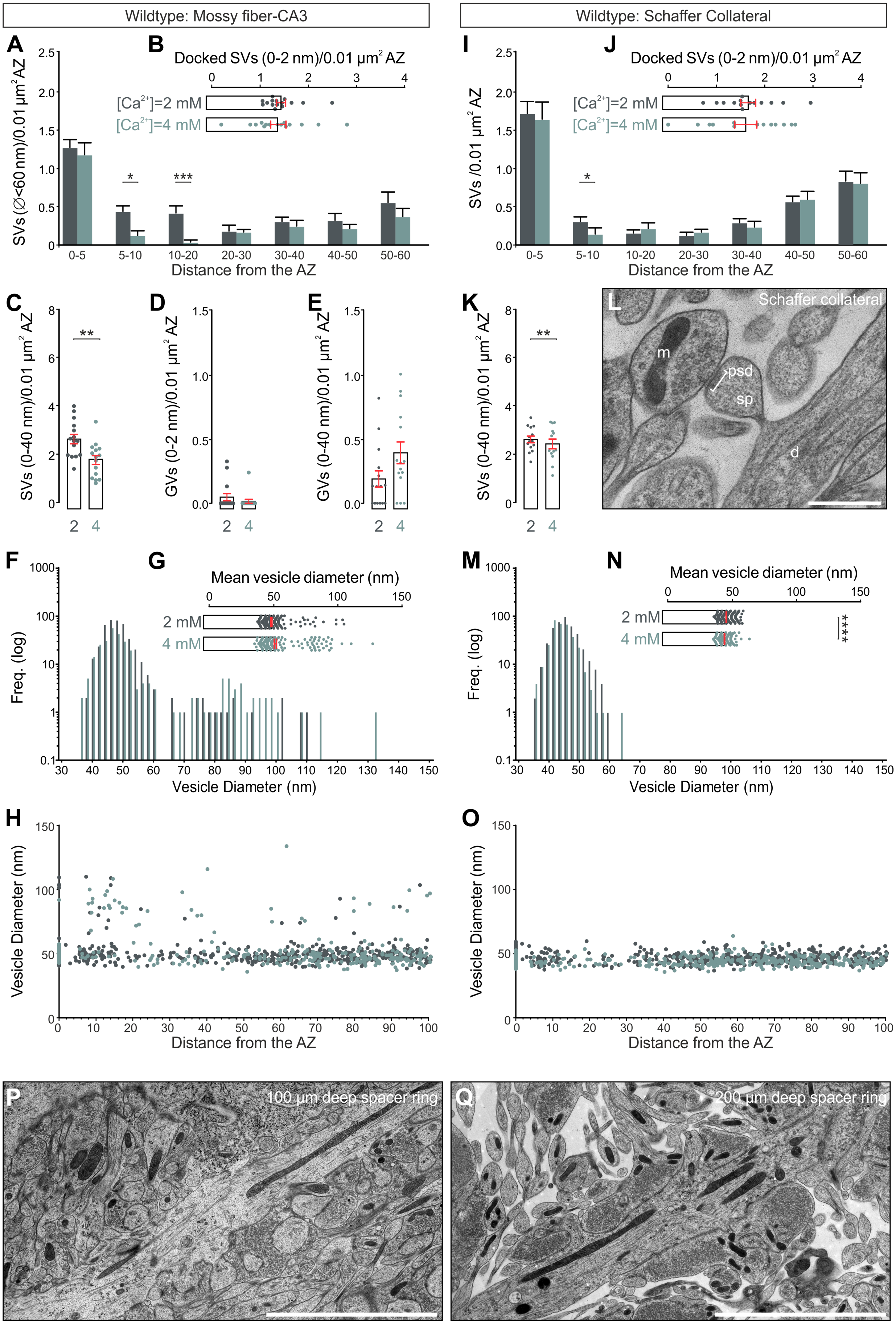

Wildtype: Mossy fiber-CA3
Wildtype: Schaffer Collateral
A
B
Docked SVs (0-2 nm)/0.01 μm² AZ
[Ca²⁺]=2 mM
[Ca²⁺]=4 mM
SVs (∅<60 nm)/0.01 μm² AZ
Distance from the AZ
I
J
SVs /0.01 μm² AZ
C
D
E
K
L
Schaffer collateral
m
psd
sp
d
SVs (0-40 nm)/0.01 μm² AZ
GVs (0-2 nm)/0.01 μm² AZ
GVs (0-40 nm)/0.01 μm² AZ
F
G
M
N
Mean vesicle diameter (nm)
2 mM
4 mM
Freq. (log)
Vesicle Diameter (nm)
H
O
Vesicle Diameter (nm)
Distance from the AZ
P
100 μm deep spacer ring
Q
200 μm deep spacer ring

Supplementary Figure 1. Comparative ultrastructural analysis of wild-type mossy fiber-CA3 and Schaffer collateral synapses at different external Ca^{2+} concentrations and in the presence of 1 µM TTX and glutamate receptor blockers in cultured hippocampal roller tube slices.

A-H, 3D Ultrastructural analysis of vesicle pools at active zones (AZs) of mossy fiber-CA3 synapses at 2 mM (dark grey) and 4 mM (green) external Ca^{2+}.

I-O, Ultrastructural analysis of vesicle pools at AZs of Schaffer collateral synapses at 2 mM (dark grey) and 4 mM (green) external Ca^{2+}.

A, **I**, Frequency distribution plotting the distribution of SVs (Ø<60 nm) within 60 nm of the AZ membrane normalized to AZ area.

B, J, Scatterplot indicating the spatial density of docked SVs (0-2 nm bin) normalized to reconstructed AZ area.

C, K, SV number within 0-40 nm of the AZ normalized to AZ area.

D, Spatial density of docked giant vesicles (GVs; Ø>60 nm) normalized to AZ area.

E, GVs within 0-40 nm of the AZ membrane normalized to AZ area.

F, M, Frequency distribution of vesicle diameters for undocked vesicles within 100 nm of the AZ membrane (2 nm bins). Mossy fiber-CA3 2mM Ca^{2+}, 420 vesicles; Mossy fiber-CA3 4mM Ca^{2+}, 273 vesicles; Schaffer collateral-CA1 2mM Ca^{2+}, 428 vesicles; Schaffer collateral-CA1 4mM Ca^{2+}, 338 vesicles.

G, N, Scatterplot indicating the mean diameter of docked vesicles. Mossy fiber-CA3 2mM Ca^{2+}, 112 vesicles; Mossy fiber-CA3 4mM Ca^{2+}, 76 vesicles; Schaffer collateral-CA1 2mM Ca^{2+}, 140 vesicles; Schaffer collateral-CA1 4mM Ca^{2+}, 109 vesicles.

H, O, Scatterplot indicating the spatial distribution of vesicles with respect to vesicle diameter.

L, Transmission electron micrograph of a Schaffer collateral synapse in the CA1 region of the hippocampus (d, dendrite; m, mitochondrion; psd, postsynaptic density; sp, spine).

P, **Q**, Electron micrographs acquired in CA3 *stratum lucidum* in which the effect of tissue compression can be appreciated in slices from the same culture prepared by high-pressure freezing using sapphire discs separated by spacer rings of 100 µm (**P**) or 200 µm (**Q**) depth.

Scale bar: **L**, 500 nm; **P** and **Q**, 5 µm. Error bars indicate mean ± SEM; $*p < 0.05$; $**p < 0.01$; $***p<0.001$; $****p < 0.0001$. Mossy fiber-CA3 2mM Ca^{2+}, 2 cultures, 2 slices, n=15 AZs; Mossy fiber-CA3 4mM Ca^{2+}; 2 cultures, 2 slices, n=15 AZs; Schaffer collateral-CA1 2mM Ca^{2+}, 2 cultures, 2 slices, n=14 AZs; Schaffer collateral-CA1 4mM Ca^{2+}, 2 cultures, 2 slices, n=13 AZs.

See also Table S1A and S1B and Figure 1.

Figure S2

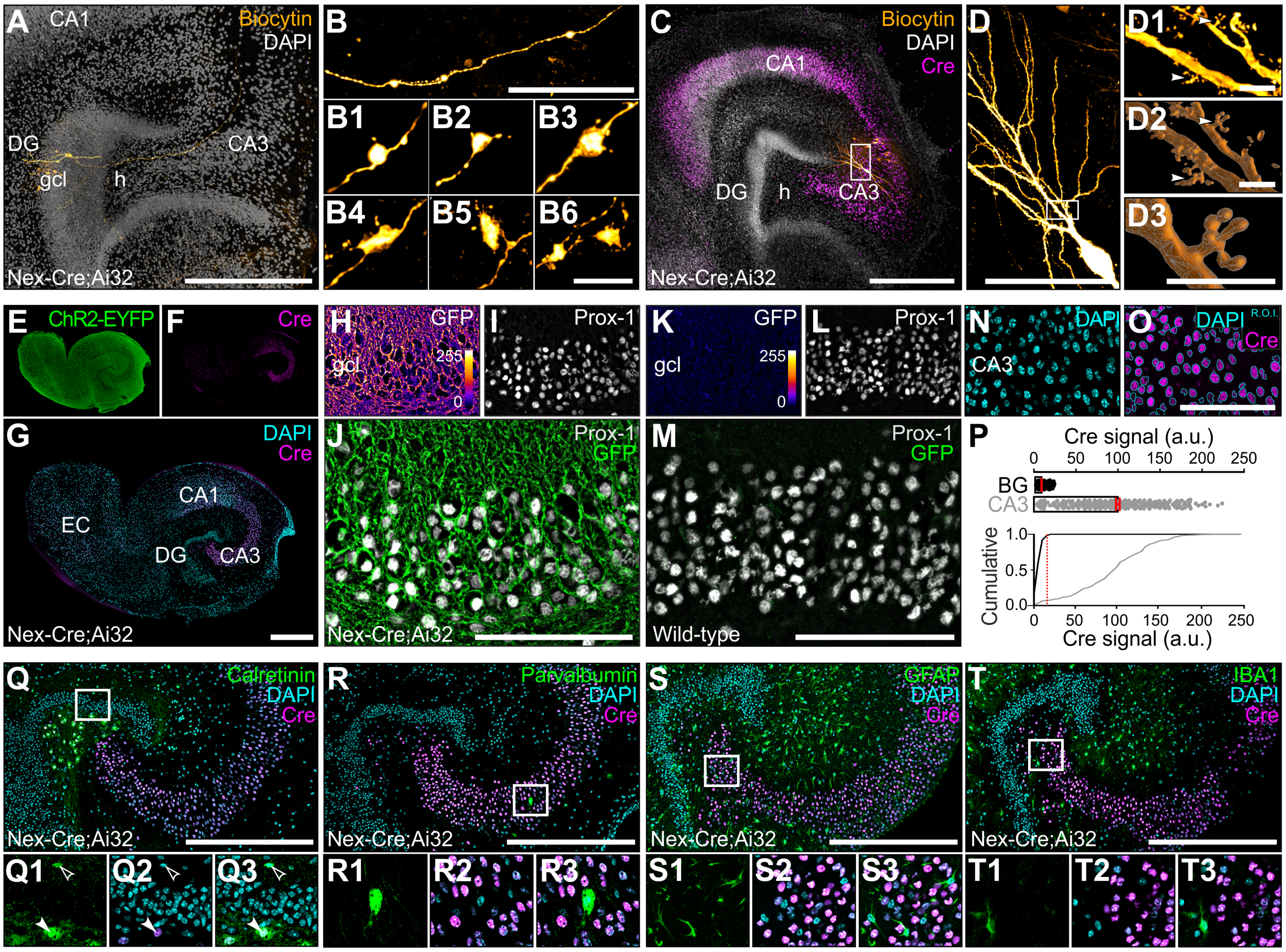

A
CA1
Biocytin
DAPI
DG
gcl
h
CA3
Nex-Cre;Ai32
B
B1
B2
B3
B4
B5
B6
C
Biocytin
DAPI
Cre
CA1
DG
h
CA3
Nex-Cre;Ai32
D
D1
D2
D3
E
ChR2-EYFP
F
Cre
H
GFP
gcl
255
0
I
Prox-1
K
GFP
gcl
255
0
L
Prox-1
N
DAPI
CA3
O
DAPI
R.O.I.
Cre
G
DAPI
Cre
CA1
EC
DG
CA3
Nex-Cre;Ai32
J
Prox-1
GFP
Nex-Cre;Ai32
M
Prox-1
GFP
Wild-type
P
Cre signal (a.u.)
0 50 100 150 200 250
BG
CA3
Cumulative
1.0
0.5
0.0
Q
Calretinin
DAPI
Cre
Nex-Cre;Ai32
R
Parvalbumin
DAPI
Cre
Nex-Cre;Ai32
S
GFAP
DAPI
Cre
Nex-Cre;Ai32
T
IBA1
DAPI
Cre
Nex-Cre;Ai32
Q1
Q2
Q3
R1
R2
R3
S1
S2
S3
T1
T2
T3

Supplementary Figure 2. Light microscopic analysis of pre- and postsynaptic development of the mossy fiber-CA3 synapse and ChR2 expression in cultured Nex-Cre;Ai32 slices. Related to Figure 1.

A, Maximum projection confocal stack in which post-hoc labelling of an electrophysiologically characterized and biocytin-filled granule cell, reveals the extent of its mossy fiber axonal projection throughout CA3 *stratum lucidum* (orange). Relative positions of dentate gyrus (DG) *stratum granulosum* and CA3 *stratum pyramidale* are shown by nuclear DAPI co-labelling (grey).

B, High magnification confocal z-stacks acquired in *stratum lucidum* reveal large and small *en passant* axonal varicosities (**B**). Large mossy fiber boutons (**B1**-**B6**) exhibit *in vivo*-like complexity, including fine filopodial extensions.

C, **D,** Maximum projection confocal stack of a post-hoc immunolabelling of an electrophysiologically characterized and biocytin-filled CA3 pyramidal neuron co-labelled for Cre-recombinase (magenta) and DAPI (grey). In *stratum lucidum*, proximal dendrites of the biocytin-filled cell (**D**, white box enlarged in **D1**) are decorated by complex, multi-compartmental spines (white arrowheads) as visualized in 3D reconstructions (**D2** and **D3**).

E-**G**, Immunohistochemical labelling against GFP detecting ChR2-EYFP (**E**, green), nuclear Cre-recombinase (**F**, **G**, magenta) and DAPI (**G**, cyan) in a Nex-Cre;Ai32 cultured slice. Nuclear Cre-recombinase signal is restricted to the *stratum pramidale*.

H-**M**, Comparative immunohistochemical analysis of membrane-targeted ChR2-EYFP signal (**H**, **K**, 'fire LUT) distribution within Prox-1 labelled (**I**, **L**, grey) granule cells of the denate gyrus in Nex-Cre;Ai32 (**H-J**) and wild-type (**K**-**M**) organotypic slices. In line with earlier findings (Goebbels et al., 2006), granule cells (GCs) of the Nex-Cre knock-in mouse line only transiently express Cre-recombinase and therefore exhibit robust ChR2-EYFP fusion protein expression (**B**), but lack Cre-recombinase immunoreactivity.

N-**P**, In the CA3 pyramidal cell layer, the vast majority of DAPI stained nuclei (**N**, cyan; **O**, cyan outline) are immunoreactive for Cre-recombinase (**O**, magenta). Scatterplot (**P**, upper) and cumulative distribution (**P**, lower) representing the quantification of nuclear Cre-recombinase signal intensity in non-principal cells (BG, 'background signal') and CA3 pyramidal cells (CA3; 'real signal') in Nex-Cre;Ai32 slice cultures.

Approximately 93.7% of all CA3 pyramidal cell nuclei have above-threshold (red dashed line) Cre-recombinase expression (**P**, BG, n = 5 slices; 9.3 a.u. ± 1.29; CA3, n = 5; 99.3 a.u. ± 2.34). **Q**-**T**, Cre-recombinase labelling (magenta) colocalizes with DAPI-positive nuclei (cyan) in calretinin-positive hilar mossy cells (**Q, Q1-Q3**, green, white arrowhead), but is not detectable in

calretinin-positive interneurons (**Q**, **Q1-Q3**, green, open arrowhead) in the molecular layer of the dentate gyrus, parvalbumin-positive interneurons (**R**, **R1-R3**, green), GFAP-positive astrocytes (**S**, **S1-S3**, green), or in IBA1-positive microglia (**T**, **T1-T3**, green).

Abbrev.: EC, entorhinal cortex; DG, dentate gyrus; CA3, *Cornu Ammonis* Area 3; CA1, *Cornu Ammonis* Area 1; gcl, granule cell layer). Scale bars: **A**, **C**, **G**, **Q**-**T**, 500 µm; **B**, **D**, **J**, **M**, **O**, 100 µm; **B6**, 10 µm; **D1**, 5 µm; **D2**, **D3**, 2µm. Error bars indicate mean ± SEM.

Figure S3

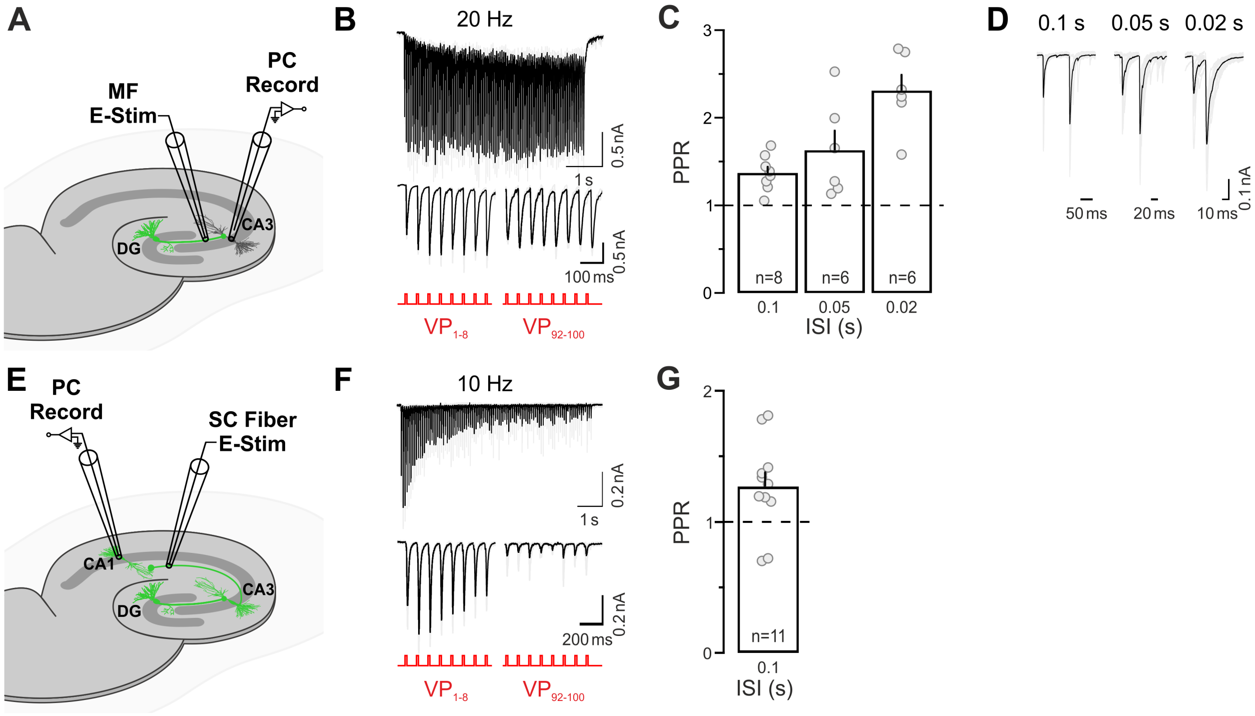

A
MF
E-Stim
PC
Record
DG
CA3
B
20 Hz
0.5 nA
1 s
0.5 nA
100 ms
VP1-8
VP92-100
C
PPR
3
2
1
0
n=8
n=6
n=6
0.1
0.05
0.02
ISI (s)
D
0.1 s
0.05 s
0.02 s
50 ms
20 ms
10 ms
0.1 nA
E
PC
Record
SC Fiber
E-Stim
CA1
DG
CA3
F
10 Hz
0.2 nA
1 s
0.2 nA
200 ms
VP1-8
VP92-100
G
PPR
2
1
0
n=11
0.1
ISI (s)

Supplementary Figure 3. Electrophysiological characterization of postsynaptic responses elicited by fiber stimulation of mossy fiber and Schaffer collateral axonal pathways in Dock10-Cre;Ai32 and Nex-Cre;Ai32 organotypic slices, respectively, via glass electrodes filled with external solution. Related to Figure 2.

A, Schematic of the experimental set-up for recording postsynaptic CA3 pyramidal cell (PC) responses to electrical fiber stimulation of the mossy fiber pathway (**B**-**D**).

B, Excitatory postsynaptic currents (EPSCs) recorded in response to 100 voltage pulses (VPs; 100 µs duration, 4 to 7 V; 20Hz) in 2 mM Ca^{2+}. Responses to the initial (lower left) and final (lower right) eight stimuli in the train are shown in the bottom panels with an expanded timescale.

C, Paired-pulse ratios measured for different inter-stimulus intervals (ISI) of 0.1 (1.37 ± 0.07, n= 8), 0.05 (1.63 ± 0.22, n= 6), and 0.02 (2.31 ± 0.18, n= 6) seconds.

D, Example traces of EPSCs recorded in response to paired pulses delivered with different inter-stimulus interval (ISI) of 0.1 or 0.05 and 0.02 seconds

E, Schematic of the experimental set-up for recording postsynaptic CA1 pyramidal cell (PC) responses to electrical fiber stimulation of the Schaffer collateral pathway (**F**-**G**).

F, EPSCs recorded in response to 100 VPs (10Hz) in 2 mM Ca^{2+}. Responses to the initial (lower left) and final (lower right) eight stimuli in the train are shown in the bottom panels at an expanded timescale.

G, Paired-pulse ratios measured for an interstimulus interval (ISI) of 0.1 second (PPR 1.27 ± 0.10, n= 11).

Error bars indicate mean ± SEM.

Figure S4

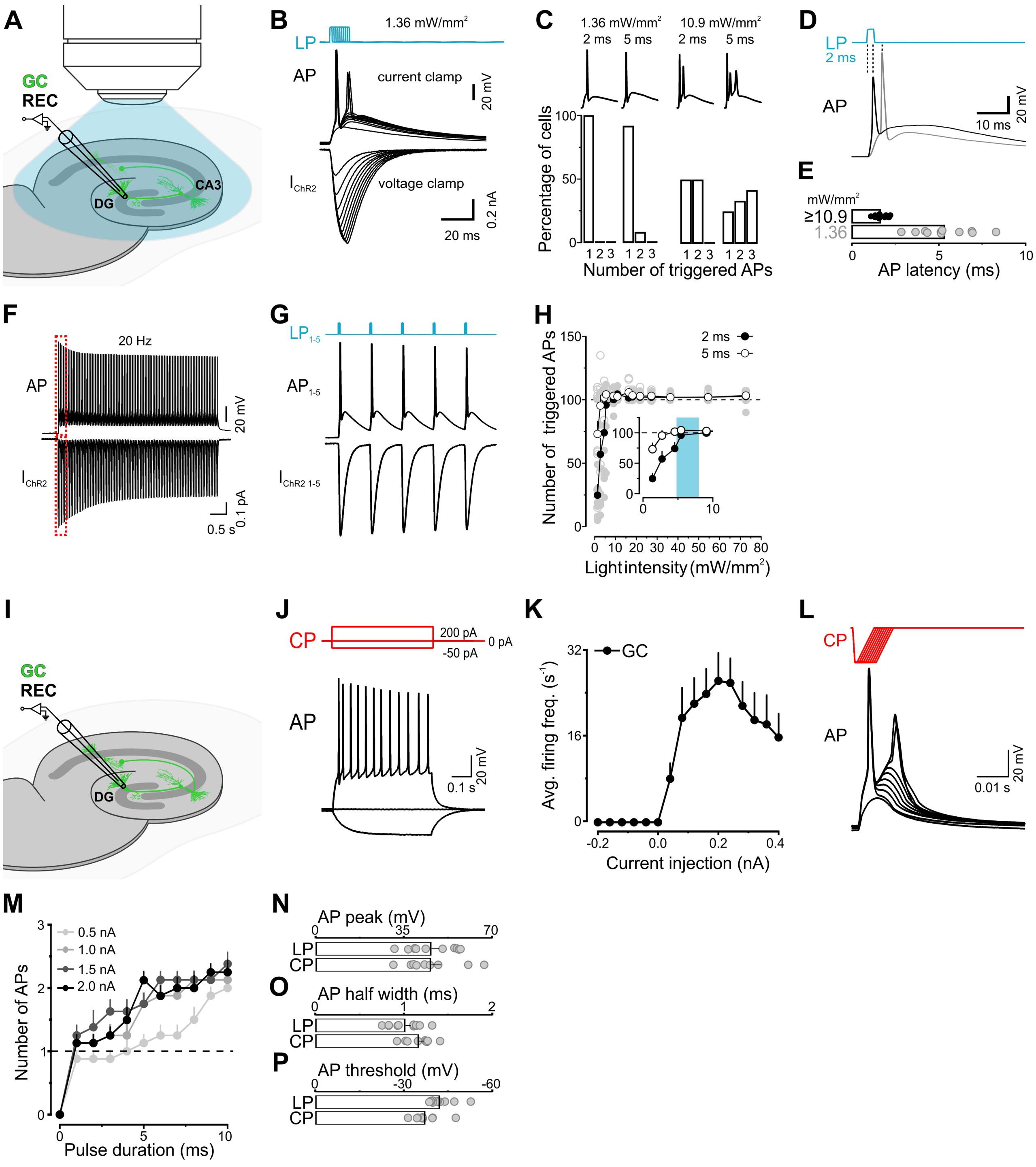

A
GC
REC
DG
CA3
B
LP
1.36 mW/mm2
AP
current clamp
20 mV
ChR2
voltage clamp
0.2 nA
20 ms
C
1.36 mW/mm2
10.9 mW/mm2
2 ms
5 ms
Percentage of cells
Number of triggered APs
D
LP
2 ms
AP
10 ms
20 mV
E
mW/mm2
≥10.9
1.36
AP latency (ms)
F
20 Hz
AP
20 mV
ChR2
0.5 s
0.1 pA
G
LP 1-5
AP 1-5
ChR2 1-5
H
2 ms
5 ms
Number of triggered APs
Light intensity (mW/mm2)
I
GC
REC
DG
J
CP
200 pA
0 pA
-50 pA
AP
0.1 s
20 mV
K
GC
Avg. firing freq. (s-1)
Current injection (nA)
L
CP
AP
0.01 s
20 mV
M
0.5 nA
1.0 nA
1.5 nA
2.0 nA
Number of APs
Pulse duration (ms)
N
AP peak (mV)
LP
CP
O
AP half width (ms)
P
AP threshold (mV)

Supplementary Figure 4. Comparative electrophysiological analysis of the properties of optically and electrically elicited action potentials in dentate gyrus granule cells in Nex-Cre;Ai32 organotypic slices. Related to Figure 2.

A, Schematic illustrating experimental configuration for analyzing AP firing properties of dentate gyrus (DG) granule cells in cultured Nex-Cre;Ai32 slices in response to light stimuli (**B**-**H**).
B, Exemplary recording of action potentials (APs) in ChR2-expressing hippocampal DG granule cells (GCs) in response to blue light (470 nm) pulses (LPs). For a given light intensity, increasing LP duration (1-10 ms) triggered zero to two APs (upper panel) and a ChR2-mediated current (I_{ChR2}) of increasing size and duration (bottom panel) in this GC.
C, Summary data on AP firing in response to LPs of either 2 ms or 5 ms duration for two light intensities (1.36 mW/mm^2 and 10.9 mW/mm^2 (n= 12 cells).
D, **E**, AP waveform (**D**) and latency (**E**) measured in response to a single 2 ms LP at two light intensities (1.36 mW/mm^2, 5.35 ± 0.49 ms, n= 11 cells; and ≥10.9 mW/mm^2, 1.67 ± 0.08 ms, n= 11 cells). Latencies are measured from the onset of the light stimulus.
F, **G**, AP discharge pattern recorded in response to 20 Hz LP trains of 5s duration (100 stimuli) (**F**). All GCs that were excitable by light intensities achievable in the high-pressure freezing chamber were also capable of faithfully following 20 Hz stimulation for at least 100 APs for a large range of light intensities. Traces exemplify an AP train recorded in current-clamp mode (upper panel) and I_{ChR2} recorded in voltage-clamp mode (lower panel) obtained in the same cell in response to 100 LPs (20 Hz, 2 ms pulse duration, light intensities of 5.45 mW/mm^2 and 1.36 mW/mm^2 for current-clamp and voltage-clamp, respectively). Note the strong inactivation of I_{ChR2} during the 5 s 20 Hz train. The initial five responses during the trains (**G**, indicated by the red dashed boxes in **F**) are shown at an expanded time scale.
H, Total number of APs triggered in GCs by 100 LP trains (20 Hz) for different light intensities and LP durations (2 ms, n= 11 cells or 5 ms, n= 10 cells). For light intensities >5 mW/mm^2, an equally high reliability of AP firing was observed in response to trains of 100 LPs of either 2 ms or 5 ms duration. For low light intensities (<5 mW/mm^2), 5 ms LP trains yielded a larger total count of APs.
I, Schematic illustrating experimental configuration for measuring AP firing properties in response to injection of depolarizing current pulses (CP) into GCs of cultured Nex-Cre;Ai32 slices (**J**-**P**).

J, Passive membrane properties and AP firing pattern in response to CP injections of ChR2-expressing hippocampal GCs were characterized under current-clamp conditions by applying families of CPs (500 ms duration, -50 pA initial amplitude, incremented in 50 pA steps).

K, GCs exhibited sustained AP-firing when injecting depolarizing CPs and had a maximum firing frequency of ~25 s^{-1} (n= 10 cells).

L, AP firing of ChR2-expressing GCs in response to depolarizing CP injections. CPs were composed of two ramps (1 ms rise, 8 ms decay) separated by a plateau of variable duration (1 - 10 ms) to mimic the shape of the ChR2-mediated current (I_{ChR2}) observed under whole-cell voltage-clamp in response to light stimulation (see Figure **S4B**).

M, Depolarizing CP injections of increasing duration and increasing amplitude (0.5 – 2 nA) triggered zero to three APs (n= 8 cells each).

N-**P**, Comparison of the properties of APs recorded in response to either stimulation by LPs or CP injections. Similar mean values were obtained for both modes of stimulation [**N**, AP peak amplitude: 46.05 ± 2.84 mV (LP, n= 11), 45.82 ± 3.81 mV (CP, n= 9); **O**, AP half width: 1.02 ± 0.05 ms (LP, n= 11), 1.17 ± 0.05 ms (CP, n= 9); **P**, AP threshold: -42.26 ± 1.26 mV (LP, n=11), -37.34 ± 1.55 mV (CP, n= 9)].

Error bars indicate mean ± SEM.

Figure S5

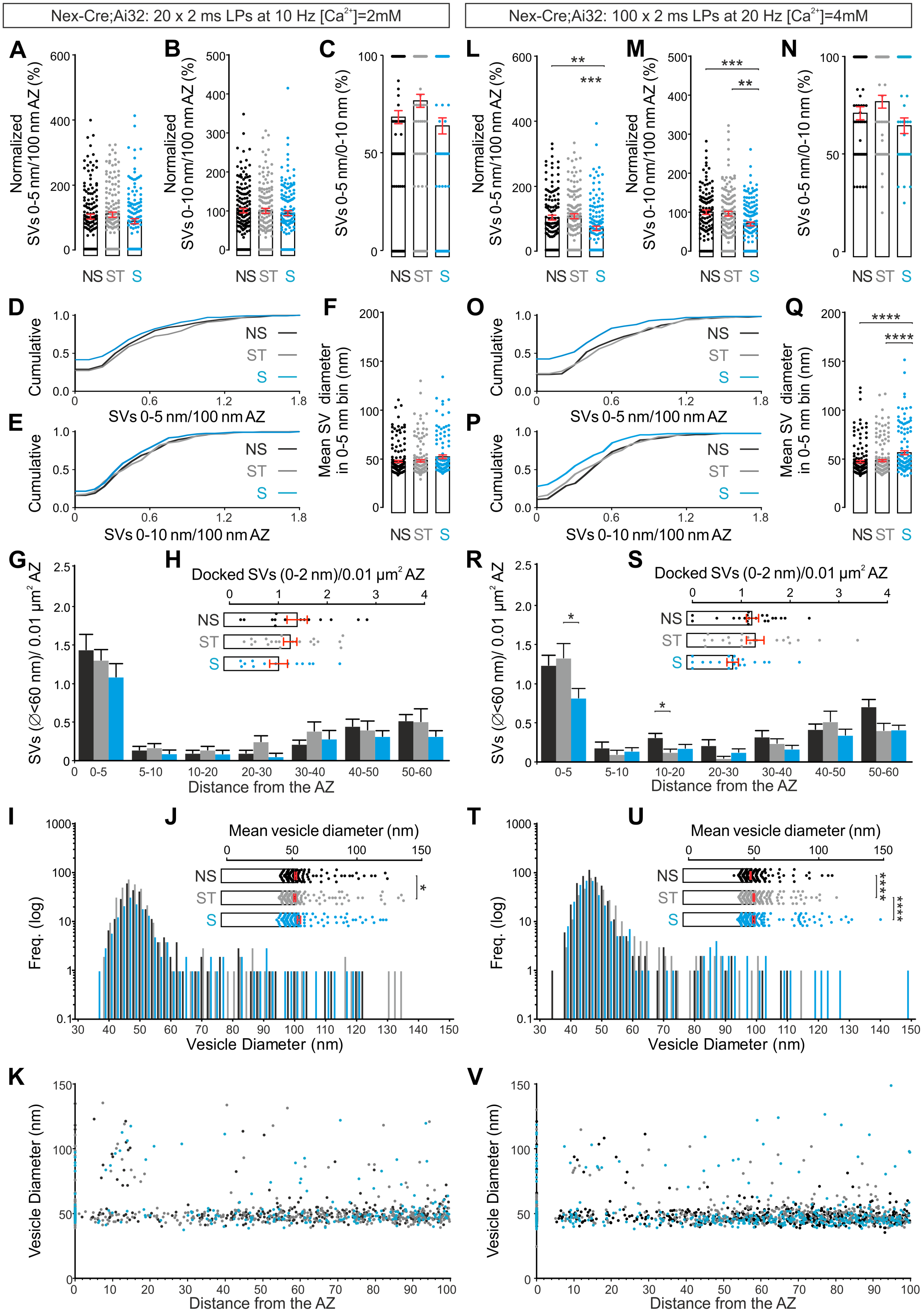

Nex-Cre;Ai32: 20 x 2 ms LPs at 10 Hz [Ca2+]=2mM
Nex-Cre;Ai32: 100 x 2 ms LPs at 20 Hz [Ca2+]=4mM
A
B
C
L
M
N
Normalized SVs 0-5 nm/100 nm AZ (%)
Normalized SVs 0-10 nm/100 nm AZ (%)
SVs 0-5 nm/0-10 nm (%)
NS ST S
**
***
D
E
F
O
P
Q
Cumulative
SVs 0-5 nm/100 nm AZ
SVs 0-10 nm/100 nm AZ
Mean SV diameter in 0-5 nm bin (nm)
****
G
H
R
S
SVs (∅<60 nm)/ 0.01 μm2 AZ
Docked SVs (0-2 nm)/0.01 μm2 AZ
0-5
5-10
10-20
20-30
30-40
40-50
50-60
Distance from the AZ
*
I
J
T
U
Freq. (log)
Mean vesicle diameter (nm)
Vesicle Diameter (nm)
K
V
Vesicle Diameter (nm)
Distance from the AZ

Supplementary Figure 5. Comparative 2D and 3D ultrastructural analyses of mossy fiber-CA3 synapses frozen immediately after mild (20 x 2 ms LPs at 10 Hz; 2 mM Ca^{2+}) or strong (100 x 2 ms LPs at 20 Hz; 4 mM Ca^{2+}) from cultured Nex-Cre;Ai32 slices.

A-**K**, 2D (**A**-**F**) and 3D (**G**-**K**) ultrastructural analyses of vesicle pools at active zones (AZs) of mossy fiber-CA3 synapses after mild stimulation. NS, no stimulation (black); ST, light stimulation + 1 µM TTX (grey); and S, light stimulation (blue).

A, **B**, Spatial density of membrane-proximal vesicles per 100 nm AZ length for vesicles within 0-5 nm (**A**) and 0-10 nm (**B**) of the AZ. Values are normalized to the no stimulation (NS) control condition.

C, Relative proportion of all vesicles within 0-10 nm of the AZ localized within in the 0-5 nm bin.

D, **E**, Cumulative frequency plots indicating the spatial distribution of membrane-proximal vesicles per 100 nm AZ length for vesicles within 0-5 nm (**D**) and 0-10 nm (**E**) of the membrane.

F, Mean diameter of vesicles within 0-5 nm of the AZ. NS, 181 vesicles; ST, 173 vesicles; S, 118 vesicles.

G, Frequency distributions plotting the distribution of SVs (Ø<60 nm) within 60 nm of the AZ membrane normalized to AZ area.

H, Spatial density of docked SVs (0-2 nm bin) normalized to reconstructed AZ area.

I, Frequency distributions of for undocked vesicles within 100 nm of the AZ membrane (2 nm bins). NS, 273 vesicles; ST, 358 vesicles; S, 175 vesicles.

J, Mean diameter of docked (0-2 nm) vesicles. NS, 95 vesicles; ST, 100 vesicles; S, 55 vesicles.

K, Spatial distribution of vesicles with respect to vesicle diameter.

L-**V**, 2D (**L**-**Q**) and 3D (**R**-**V**) ultrastructural analyses of vesicle pools at AZs of mossy fiber-CA3 synapses after strong stimulation.

L, **M**, Spatial density of membrane-proximal vesicles per 100 nm AZ length for vesicles within 0-5 nm (**L**) and 0-10 nm (**M**) of the AZ. Values are normalized to the no stimulation (NS) control condition.

N, Relative proportion of all vesicles within 0-10 nm of the AZ localized within in the 0-5 nm bin.

O, **P**, Cumulative frequency plots indicating the spatial distribution of membrane-proximal vesicles per 100 nm AZ length for vesicles within 0-5 nm (**O**) and 0-10 nm (**P**) of the membrane.

Q, Mean diameter of vesicles within 0-5 nm of the AZ. NS, 180 vesicles; ST, 197 vesicles; S, 129 vesicles.

R, Frequency distributions plotting the distribution of SVs (Ø<60 nm) within 60 nm of the AZ membrane normalized to AZ area.

S, Spatial density of docked SVs (0-2 nm bin) normalized to reconstructed AZ area.
T, Frequency distributions of for undocked vesicles within 100 nm of the AZ membrane (2 nm bins). NS, 483 vesicles; ST, 375 vesicles; S, 336 vesicles.
U, Mean diameter of docked (0-2 nm) vesicles. NS, 107 vesicles; ST, 134 vesicles; S, 88 vesicles.
V, Spatial distribution of vesicles with respect to vesicle diameter.

2D Analysis (**A**-**F**): NS, 3 cultures, 4 slices, 44 MFBs, n=144 AZs; ST, 3 cultures, 4 slices, 39 MFBs, n=131 AZs; S, 2 cultures, 3 slices, 36 MFBs, n= 135 AZs.
3D Analysis (**G**-**K**): NS, 2 cultures, 3 slices, 14 AZs; ST, 2 cultures, 3 slices, n=21 AZs; S, 1 culture, 2 slices, n= 13 AZs.
2D Analysis (**L**-**Q**): NS, 2 cultures, 2 slices, 38 MFBs, n=120 AZs; ST, 2 cultures, 2 slices, 42 MFBs, n=127 AZs; S, 2 cultures, 2 slices, 44 MFBs, n= 139 AZs.
3D Analysis (**R**-**V**): NS, 2 cultures, 2 slices, n=21 AZs; ST, 2 cultures, 2 slices, n=21 AZs; S, 2 cultures, 2 slices, n= 21 AZs.

Error bars indicate mean ± SEM; *p < 0.05; **p < 0.01; ***p<0.001; ****p < 0.0001.

See also Figures 4 and 5 and Table S1G-S1J.

Figure S6

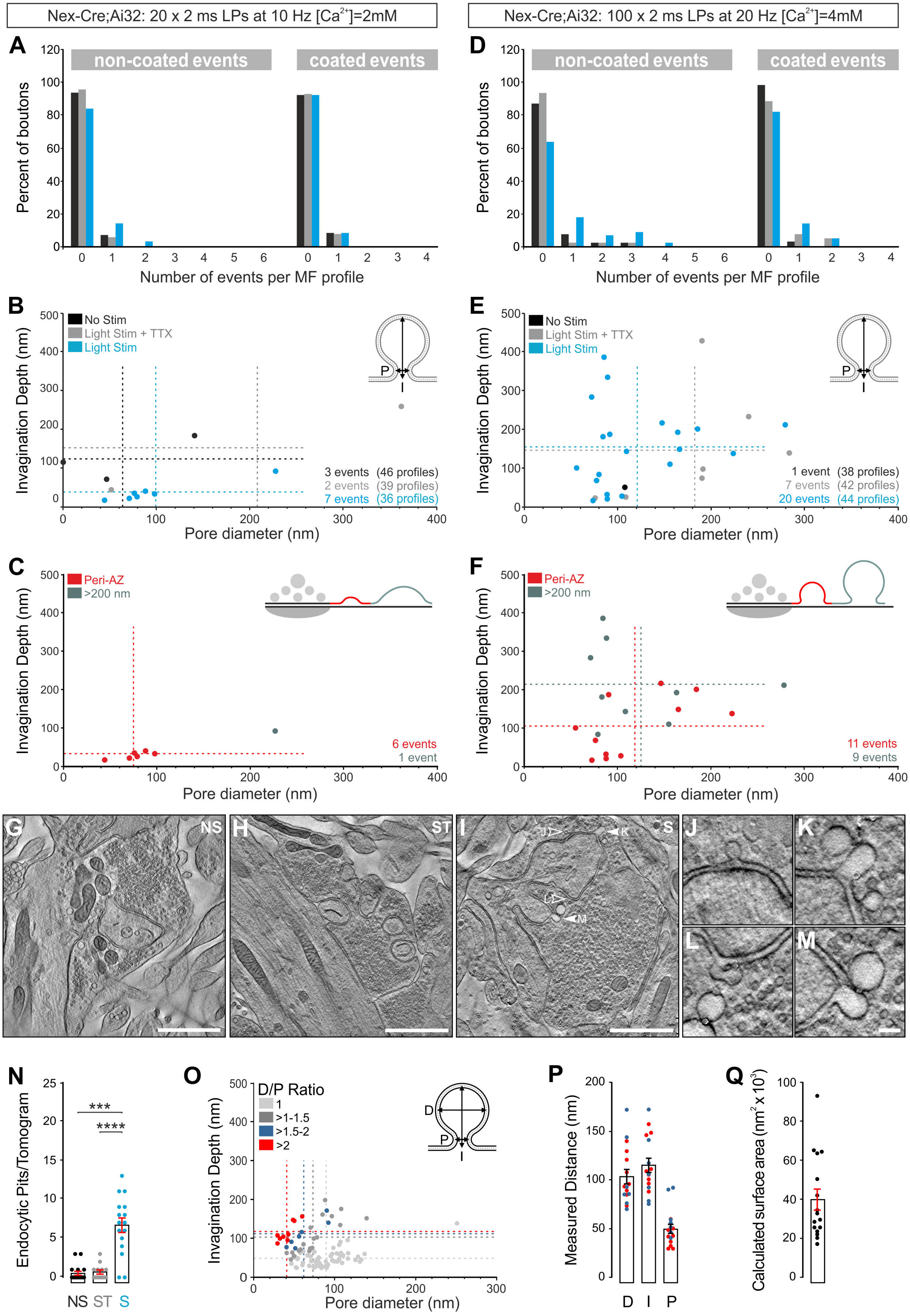

Nex-Cre;Ai32: 20 x 2 ms LPs at 10 Hz [Ca2+]=2mM
Nex-Cre;Ai32: 100 x 2 ms LPs at 20 Hz [Ca2+]=4mM
A
D
non-coated events
coated events
Percent of boutons
Number of events per MF profile
B
E
No Stim
Light Stim + TTX
Light Stim
Invagination Depth (nm)
Pore diameter (nm)
P
I
3 events (46 profiles)
2 events (39 profiles)
7 events (36 profiles)
1 event (38 profiles)
7 events (42 profiles)
20 events (44 profiles)
C
F
Peri-AZ
>200 nm
6 events
1 event
11 events
9 events
G
NS
H
ST
I
S
J
K
L
M
N
Endocytic Pits/Tomogram
***
****
NS ST S
O
D/P Ratio
1
>1-1.5
>1.5-2
>2
D
P
Measured Distance (nm)
D I P
Q
Calculated surface area (nm2 x 103)

Supplementary Figure 6. Comparative 2D and 3D ultrastructural analyses of endocytic profiles in mossy fiber-CA3 synapses frozen immediately after mild (20 x 2 ms LPs at 10 Hz; 2 mM Ca^{2+}) or strong (100 x 2 ms LPs at 20 Hz; 4 mM Ca^{2+}) from cultured Nex-Cre;Ai32 slices.

A-**C**, 2D ultrastructural analyses of endocytic profiles in mossy fiber-CA3 synapses after mild stimulation. NS, no stimulation (black); ST, light stimulation + 1 µM TTX (grey); and S, light stimulation (blue).

D-**F**, 2D ultrastructural analyses of endocytic profiles in mossy fiber-CA3 synapses after strong stimulation.

A, **D**, Histogram plotting the relative incidence of coated and non-coated membrane invaginations observed in the three experimental conditions: NS, no stimulation (black); ST, light stimulation in the presence of 1 µM TTX (grey); and S, light stimulation (blue).

B-**F**, Relationship between pore diameter (P) and invagination depth (I) for non-coated endocytic intermediates across all conditions (**B**, **E**) and for non-coated endocytic intermediates detected exclusively in stimulated mossy fiber boutons at peri-active zonal (red) and more distally localized (green) sites (**C**, **F**). Dashed lines indicate respective mean values.

G-**Q**, 3D ultrastructural analyses of endocytic profiles in mossy fiber-CA3 synapses after strong stimulation.

G-**I**, Representative electron tomographic subvolumes acquired at 5000x magnification from 350 nm-thick sections through MFBs from three experimental conditions: NS, no stimulation (**G**), ST, stimulation in the presence of 1 µM TTX (**H**), and S, stimulation (**I**).

J-**M**, Manipulation of tomographic slice tilt angles revealed the shape and full extent of individual membrane invaginations indicated in **I**.

N, Relative incidence of putative endocytic profiles per tomogram.

O, Relationship between pore diameter (P) and invagination depth (I) according to the ratio of maximum diameter (D) and pore diameter (D/P= 1, light grey; D/P= >1-1.5, dark grey; D/P= 1.5-2, blue; D/P= >2, red; respective mean values indicated by dashed lines).

P, Scatterplot indicating parameters (D, maximum diameter; I, invagination depth; P, pore diameter) pooled from endocytic events with D/P ratios of 1.5-2 (blue) and >2 (red) for pore diameters < 100 nm.

Q, Calculated surface area of predicted endocytosed vesicles from profiles with D/P ratios >1.5.

Scale bars: **G-I**, 1 μm; **M**, 100 nm. Error bars indicate mean ± SEM; ***$p<0.001$; ****$p < 0.0001$.
2D Analysis (**A-C**): NS, 3 cultures, 4 slices, 47 MFBs; ST, 3 cultures, 4 slices, 39 MFBs; S, 2 cultures, 3 slices, 36 MFBs.
2D Analysis (**D-F**): NS, 3 cultures, 4 slices, 38 MFBs; ST, 3 cultures, 4 slices, 42 MFBs; S, 2 cultures, 3 slices, 36 MFBs.
3D Analysis (**G-Q**): NS, 2 cultures, 2 slices, n=17 tomograms; ST, 2 cultures, 2 slices, n=13 tomograms; S, 2 cultures, 2 slices, n= 16 tomograms.

See also Figures 6 and 7, Table S1K-S1M and Video S2.

Figure S7

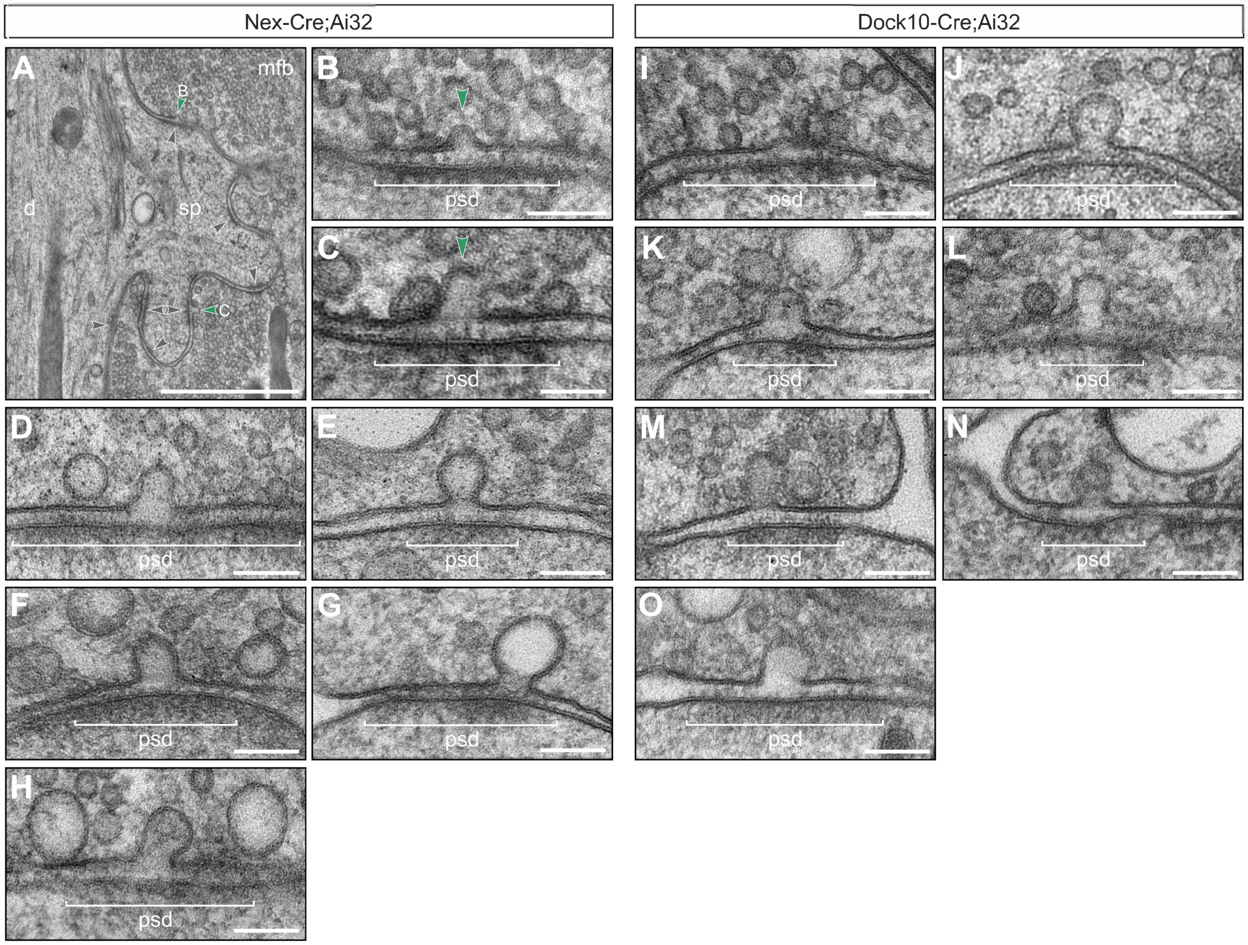

Nex-Cre;Ai32
Dock10-Cre;Ai32
A
mfb
B
d
sp
C
B
psd
C
psd
D
psd
E
psd
F
psd
G
psd
H
psd
I
psd
J
psd
K
psd
L
psd
M
psd
N
psd
O
psd

Supplementary Figure 7. Omega-profiles detected directly at mossy fiber presynaptic active zones in optically stimulated Nex-Cre;Ai32 and Dock10;Ai32 slices.

A-**C**, Active zone (AZ) localized omega profiles (green arrowheads) observed in direct apposition to the postsynaptic density (**A**, black arrowheads; **B**-**C**, psd) in stimulated Nex-Cre;Ai32 slices (20x 2 ms LPs at 10 Hz; 2 mM Ca^{2+}).
D-**H**, AZ localized omega profiles observed in direct apposition to the postsynaptic density (psd) in stimulated Nex-Cre;Ai32 slices (100 x 2 ms LPs at 20 Hz; 4 mM Ca^{2+}).
I-**O**, AZ localized omega profiles observed in direct apposition to the postsynaptic density (psd) in stimulated Dock10-Cre;Ai32 slices (100 x 5 ms LPs at 20 Hz; 2 mM Ca^{2+}).

Abbrev.: d, dendrite; mfb, mossy fiber bouton; sp, complex spine; psd, postsynaptic density. Scale bars: **A**, 1 µm; **B**-**O**, 100 nm.

See also Figure 6.
